# Controlled Twill Surface Structure Endowing Nanofiber Composite Membrane Excellent Electromagnetic Interference Shielding

**DOI:** 10.1007/s40820-024-01444-y

**Published:** 2024-07-04

**Authors:** Dechang Tao, Xin Wen, Chenguang Yang, Kun Yan, Zhiyao Li, Wenwen Wang, Dong Wang

**Affiliations:** 1 Key Laboratory of Textile Fiber and Products (Wuhan Textile University), Ministry of Education, Wuhan Textile University, Wuhan, 430200 People’s Republic of China; 2 College of Chemistry, Chemical Engineering and Biotechnology, Donghua University, Shanghai, 201620 People’s Republic of China

**Keywords:** Twill surface structure, MXene/AgNW, Nanofiber membrane, Electromagnetic interference, Flexibility and mechanical properties

## Abstract

Inspired by the Chinese Knotting weave structure, an electromagnetic interference (EMI) nanofiber composite membrane with a twill surface was prepared. Poly(vinyl alcohol-co-ethylene) (Pva-*co*-PE) nanofibers and twill nylon fabric were used as the matrix and filter templates, respectively. A Pva-co-PE-MXene/silver nanowire (Pva-co-PE-MXene/AgNW, PM_x_Ag) membrane was successfully prepared using a template method. When the MXene/AgNW content was only 7.4 wt% (PM_7.4_Ag), the EMI shielding efficiency (SE) of the composite membrane with the oblique twill structure on the surface was 103.9 dB and the surface twill structure improved the EMI by 38.5%. This result was attributed to the pre-interference of the oblique twill structure in the direction of the incident EM wave, which enhanced the probability of the electromagnetic waves randomly colliding with the MXene nanosheets. Simultaneously, the internal reflection and ohmic and resonance losses were enhanced. The PM_7.4_Ag membrane with the twill structure exhibited both an outstanding tensile strength of 22.8 MPa and EMI SE/t of 3925.2 dB cm^−1^. Moreover, the PM_x_Ag nanocomposite membranes demonstrated an excellent thermal management performance, hydrophobicity, non-flammability, and performance stability, which was demonstrated by an EMI SE of 97.3% in a high-temperature environment of 140 °C. The successful preparation of surface-twill composite membranes makes it difficult to achieve both a low filler content and a high EMI SE in electromagnetic shielding materials. This strategy provides a new approach for preparing thin membranes with excellent EMI properties.

**Supplementary Information:**

The online version contains supplementary material available at 10.1007/s40820-024-01444-y.

## Highlights

- Inspired by the Chinese Knotting weave structure, an electromagnetic interference (EMI) nanofiber composite membrane with a twill surface was prepared.
- The EMI shielding efficiency (SE) of the composite membrane was 103.9 dB when the MXene/silver nanowires (MXene/AgNW) content was only 7.4 wt% and the surface twill structure improved the EMI by 38.5%.
- The nanofiber composite membrane demonstrated an excellent thermal management performance, hydrophobicity, non-flammability, and performance stability, which was demonstrated by an EMI SE of 97.3% in a high-temperature environment at 140 °C.

**Supplementary Information:**

The online version contains supplementary material available at 10.1007/s40820-024-01444-y.

## Graphical abstract

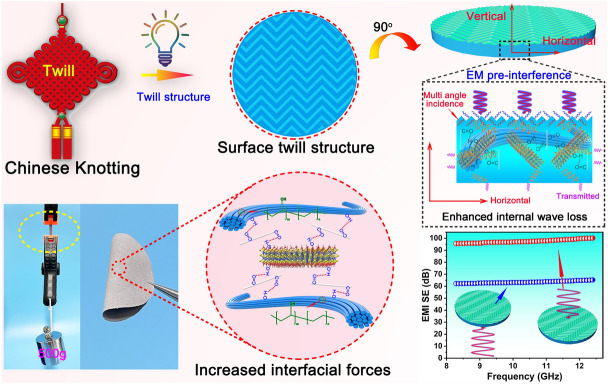

## Introduction

The development of wireless technology and widespread use of highly integrated electronic equipment have significantly improved the living quality; but they have also led to unnecessary electromagnetic radiation in the surrounding environment [1–5]. This electromagnetic pollution can lead to the failure and damage of critical electronic components [6, 7]. Excessive electromagnetic radiation has harmful effects on human health and seriously affects the living quality. Therefore, high-performance, flexible, controllable, and efficient electromagnetic interference shielding materials are important for solving these urgent problems [8–10].

MXene, a new two-dimensional transition metal carbide/nitride material, has gained widespread attention for the construction of a high-performance EMI [2, 3, 11–13]. However, MXene membranes typically exhibit poor mechanical properties and severe structural degradation in humid and aqueous environments [14, 15]. To improve the mechanical properties and hydrophobicity of the MXene-based composites, MXene nanosheets were mixed with various polymer matrix or adhesives by interfacial interactions [2, 16, 17]. Commonly used substrates or assemblies include melamine foams [3, 18], poly(3, 4-ethyldioxythiophene)-poly (styrene sulfonate) [19], carbon materials [20], polyurethanes [21], xanthan gum [22], cellulose nanofibers (CNF) [23], and arylamine nanofibers (ANF) [24, 25]. A variety of hybrid systems using MXenes as the absorbing medium have been studied, reporting that a high absorption performance and excellent mechanical strength can be simultaneously achieved [4, 16, 17].

Excellent EMI efficiency results have been obtained using various material types and structures, including the design of hybrid systems and construction of special structures [26–28]. Hybrid systems require considering the mechanisms of impedance-matching loss between the dielectric and mixed materials. Shahazed et al. [14] prepared MXene membranes by vacuum filtration, and those with a thickness of 45 μm achieved a high transmission loss value of 92.0 dB. Ma et al. [29] prepared a two-layer ANF–MXene/AgNW nanocomposite paper using a simple two-step vacuum-assisted filtration and hot-pressing method. Using a 20 wt% content, the composite paper exhibited an excellent EMI shielding efficiency (SE) of 48.1 dB. Jiao et al. [30] prepared MXene/water-based polyurethane/natural rubber composite membranes by combining electrostatic repulsion and vacuum-assisted filtration. In the frequency range of 8.2–40.0 GHz, the sound transmission loss (SET) was 76.1 dB. Gu et al. [2] successfully prepared a (Fe_3_O_4_/PI)–Ti_3_C_2_T_x_–(Fe_3_O_4_/PI) composite membrane using PI as the matrix and PMMA microspheres as the template via a sacrificial template method based on the design of a porous/multilayer structure; the highest EMI SE value was 85 dB. Ma et al. [31] first prepared Ti_3_C_2_T_x_/Polydopamine (PDA)-polyethyleneimine (PEI)@PP particles using a flocculation method. The Ti_3_C_2_T_x_/PDA-PEI@PP composite exhibited a maximum SET of 78.85 dB. Lu et al. [32] prepared MXene/ANF hybrid aerogels with a microporous structure. The MXene/ANF hybrid aerogel exhibited an SET of 56.8 dB, with an EMI SSE of 3645.7 dB cm^2^ g^−1^. Utilizing a copper-coated polydimethylsiloxane method, He et al. [33] were able to improve electromagnetic shielding to a level of 30.9 dB under pre-stretching conditions. Liang et al. [34] developed a flexible solid–solid phase change material (PCM) coating using a self-crosslinking polyethylene glycol (PEG) (IPTS) reaction. The modified textile exhibited an electromagnetic interference shielding effect of approximately 72 dB when the thickness was 0.26 mm.

Considering the rapid development of portable, intelligent, multifunctional, and wearable electronic devices, composite membranes with design flexibility and excellent toughness have received widespread attention in the field of EMI [7, 29, 35, 36]. Tan et al. [37] prepared MXene/CS composite membranes using a layer-by-layer self-assembly strategy. The results demonstrated that when the mass of MXene was 25.9 wt%, the EMI SE of the 35 μm thick MXene/CS membrane reached 40.8 dB. Jin et al. [38] successfully prepared MXene/PVA composite membranes with multilayer alternating structures via layer-by-layer casting. The results demonstrated that the EMI SE of the MXene/PVA membrane with a thickness of 27 μm and MXene content of 19.5 wt% reached 44.4 dB. Several studies have been conducted to achieve an efficient electromagnetic interference shielding performance by using mixed systems and special structures with MXene dielectrics [5, 14, 32, 37]. However, the expensive and high dielectric filling levels of the current-absorbing media employed for electromagnetic shielding may have an impact on the mechanical characteristics of the composite membranes. Notably, the goal of reducing the number of absorbing media and improving the electromagnetic shielding performance are contradictory. Therefore, the preparation of composite membranes that are flexible, low in cost, and demonstrate a highly efficient electromagnetic interference shielding performance is an urgent challenge. The development of textile-based electromagnetic shielding materials has recently become a popular research topic [16, 33, 34]. However, electromagnetic shielding materials based on textile fabrics may have limitations in new structural designs, performance stability, textile flexibility attenuation, and internal hybrid system construction [39–41]. In summary, the conventional superposition of different media is difficult to solve the problem of the preparation of thin membranes with flexible and high electromagnetic shielding properties, and it is known that the added conductive media is often more expensive, which greatly limits the application of such materials. Therefore, the development of composite membranes with flexibility, high electromagnetic shielding performance, and low cost needs to start with the design of new structures and reduction in the dielectric filling amount. Polyvinyl alcohol-polyethylene copolymer (Pva-co-PE) nanofibers as substrates can attain the flexible design of a variety of structures, have good flexibility, and are an ideal matrix for the preparation of flexible materials with high electromagnetic shielding properties [42–46].

Inspired by the Chinese Knotting structure, a twill structure was constructed on the surface of a composite membrane for the first time to achieve pre-interference in the direction of the incident electromagnetic wave, enhancing the electromagnetic wave interface reflection and internal wave loss, and improving the EMI SE of the composite membrane. The PM_x_Ag nanofiber composite membrane with a twill surface was prepared in one step by vacuum filtration combined with the template method. Nanofiber membranes with a high EMI SE were obtained by the enhanced surface reflection and internal loss of the EM waves, proving that a twill structure with a special surface can be used to predisturb the incident direction of the EM waves, enabling the incident wave to enter the absorbing composite membrane at various angles and significantly enhancing the loss of EM waves in the internal transmission. The introduction of the EM wave pre-interference mechanism differs from the high-efficiency absorption mechanism of traditional hybrid systems, and the low amount of filling of the absorbing medium significantly reduces the preparation cost while retaining the advantageous super-flexibility of the nanofiber membrane. Both experimental and theoretical optimization calculations revealed that the synergistic effects of the pre-interference structure, porous structure of the internal space, and matching of the absorbing media are the key reasons for producing this impressive EMI SE. Our results demonstrated that this twill structure played a critical role in the attenuation process of the EM waves. Moreover, the obtained composite membrane simultaneously achieved improvements in the strength, flame retardancy, thermoelectric conversion, and hydrophobicity. These excellent comprehensive properties introduce a new strategy for constructing a pre-interference structure in the practical application of EMI shielding.

## Experimental Section

Main materials and characterization methods are provided in the Supporting Information.

### Preparation of PM_x_Ag Nanofiber Membranes with Twill Structure

**Fig. 1:**
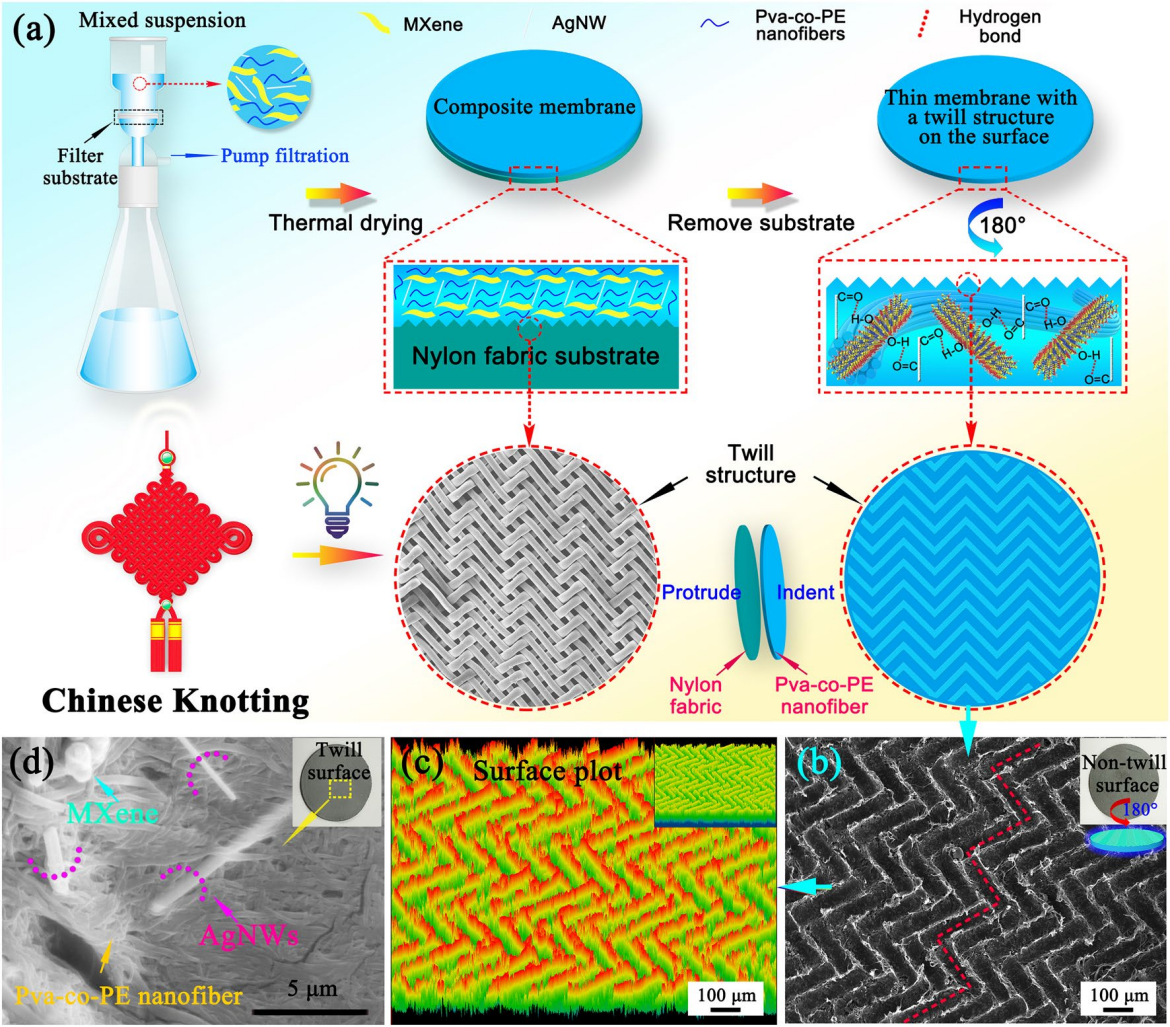
**a** Schematic illustration for the fabrication of the composite nanofiber membrane with the pre-interference twill structure. **b** SEM image of the twill surface of the composite nanofiber membrane. **c** Surface plot of the twill surface. **d** Magnified SEM image of the twill surface

Depending on the desired final MXene/AgNW content, a uniform mixture of Ti_3_C_2_T_x_ MXene and AgNW dispersion was obtained by ultrasound and agitation and then added proportionally to the prepared Pva-co-PE nanofiber suspension for secondary dispersion. In the filtration process, the nylon fabric with twill structure was used as the substrate. After the filtration, the membranes were dried and then stripped the nylon fabric to obtain the Pva-co-PE composite membrane with twill structure on the surface. A series of composite membranes with MXene/AgNW content of 1.6, 2.2, 5.2, and 7.4 wt% were prepared. In these cases, the weight ratio of Ti_3_C_2_T_x_ to AgNW was set to 10:1. For comparison, the Pva-co-PE nanofiber membrane was prepared using the same method. Based on the MXene/AgNW content, the composite membranes were labeled PM_1.6_Ag, PM_2.2_Ag, PM_5.2_Ag, and PM_7.4_Ag. The preparation process of the composite membrane is illustrated in Fig. 1a.

### Material Characterizations

The morphologies of MXene, AgNW, and PM_x_Ag membranes were characterized by scanning electron microscopy (SEM, JEOL JSM-IT300, Japan) and optical microscopy (Zeiss, Primotech), respectively. Fourier spectrometer (Shimadzu, Tracer-100), X-ray photoelectron spectrometer (XPS, Thermo ESCALAB 250XI), X-ray diffractometer (XRD, empyrean), thermogravimetric analyzer (Netzsch, TG 209 F1 Tarsus), and Ultraviolet absorber (Shimadzu, Solidspec-3700) were used to collect the chemical structure information, elemental composition, crystal diffraction peak data, thermal stability and UV absorption coefficient, respectively. A 3D X-ray microscope (Xradia, 515 Versa, Zeiss) was used to observe the surface structure of the composite membrane and the distribution of the MXene/AgNW. The electrical conductivity, surface temperature change, cyclic compression, tensile and hydrophobic properties of the materials were measured by four-probe tester (RTS-4), infrared imaging device (Fluke, Ti 400, Everett, USA), cyclic compression dynamometer (Mark-10, Model M5-10), tension machine (Instron 5936) and attension theta system (KSV Instruments Ltd., Finland). High-temperature electromagnetic shielding test: The PM_5.2_Ag composite membrane was heated by thermoelectric conversion at 4 V, and its surface temperature was measured using a fluke infrared imaging device. The EMI performance was immediately detected when the temperature was raised to 60, 80, 100, 120, and 140 °C, respectively. The EMI SE coefficient was calculated using Eq. 1.

1 $$EMI\,SE\,coefficient\left( \% \right) = \frac{{EMI\,SE_{{high\,temperature}} }}{{EMI\,SE_{{room\,temperature}} }} \times 100$$

A new manual infrared tablet press (Tianjin Botian Shengda Technology Development Co., LTD, FW-5A) was used to test the compression properties of the twill structure on the surface of the composite membrane at compressive stresses of 2.5, 5.0, and 10 MPa. Surface SEM morphology observation, electromagnetic shielding performance, and water contact angle test analyses were then performed.

## Results and Discussion

### Preparation and Microstructure of the PM_x_Ag Nanofiber Composite Membrane

A schematic of the preparation of the PM_x_Ag composite membranes is shown in Fig. 1a. Inspired by the structure of Chinese Knotting (Fig. 1a), a twill braided nylon fabric was used as the extraction filter base, and the nylon fabric was removed after the extraction filtration drying treatment (Fig. S3). A structure similar to that of Chinese Knotting was formed on the PM_x_Ag composite surface (template method). An SEM image of the surface structure of the PM_x_Ag membrane is shown in Fig. 1b. The surface of the composite membrane formed a conventional structure similar to that of a concave twill. As shown in the surface topography in Fig. 1c, the surface demonstrated a high regularity and significant gradient distribution. Figure 1d presents the twill-surface topography of the prepared composite membrane, which clearly demonstrates that the Ag nanowires and MXene nanosheets were composites in the Pva-co-PE nanofiber membrane. Figure S5 presents SEM images of the twill structure on the surface of the nanofiber composite membranes with different component ratios. The twill structures of the PM_x_Ag composite membranes exhibit good stability. In addition, the enlarged images of the MXene/AgNW show a trapezoidal distribution on the surface of the membranes along the twill structure (Fig. S5a), whereas the topography height image demonstrates that the MXene/AgNW are progressively distributed high and low along the twill structure (Fig. S5a). Figure S6 shows magnified SEM images of the surfaces of the PM_5.2_Ag composite membranes. The MXene/AgNW exhibited a trapezoidal distribution owing to the extrusion of the template twill nylon fabric during extraction.

### Structural Analysis of the PM_x_Ag Nanofiber Composite Membrane

**Fig. 2:**
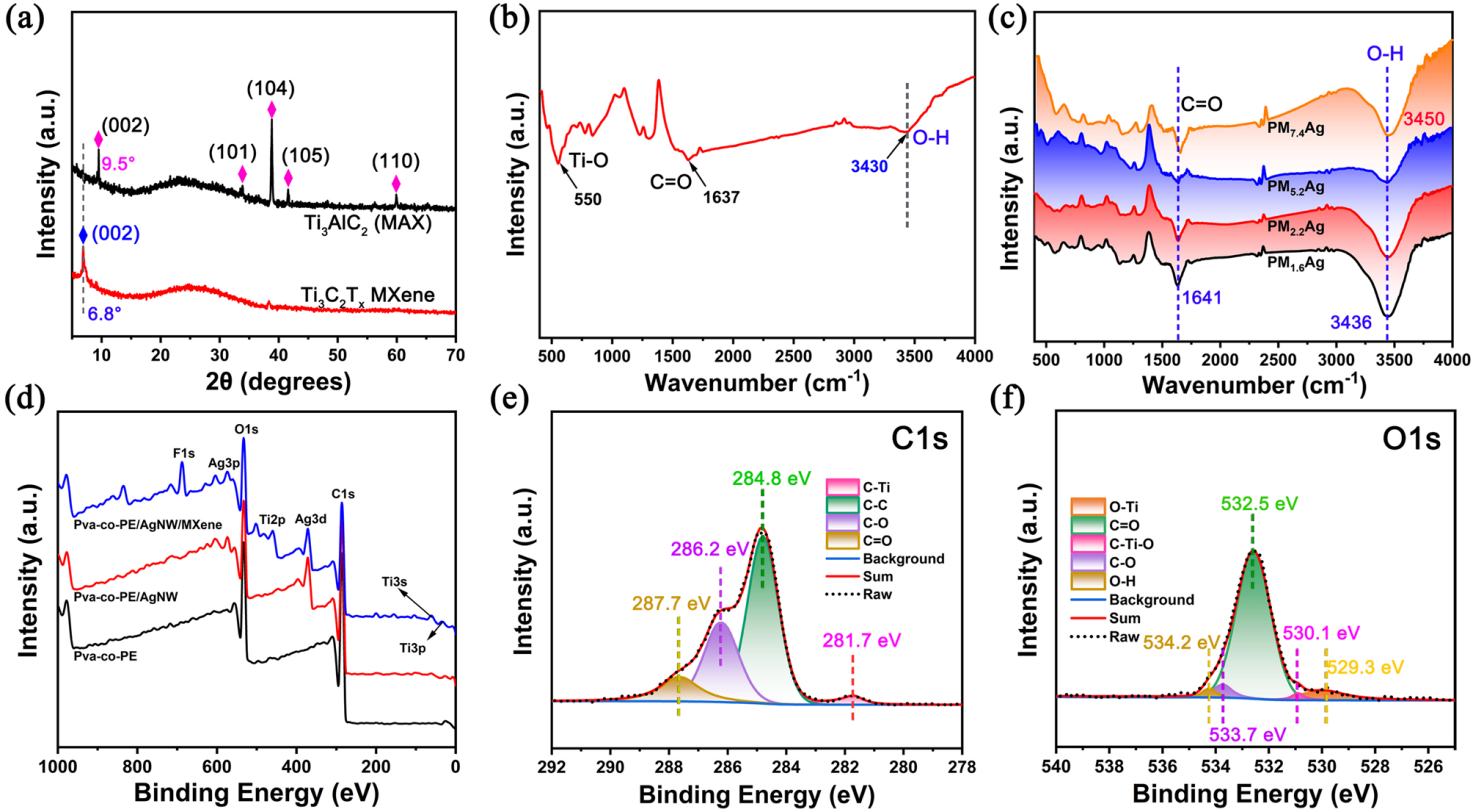
**a** XRD patterns of the of MAX and MXene. **b, c** FTIR spectra of the Ti_3_C_2_T_x_ MXene and PM_x_Ag composite membranes. **d** XPS wid-scan spectra of PM_x_Ag composite membranes. **e, f** High-resolution XPS spectra of C 1*s*, O 1*s* for the PM_x_Ag composite membranes

As shown in Fig. 2a, the peak corresponding to the (104) plane of Ti_3_AlC_2_ (MAX) nearly disappeared after etching and exfoliation, confirming that the Al layer in the parent Ti_3_AlC_2_ (MAX) phase was removed [47]. In addition, the (002) peak of Ti_3_C_2_T_x_ MXene at 9.5° shifted to a lower 2θ angle of 6.8°. The left shift of the (002) peak indicates an increase in the *d*-spacing owing to the removal of the Al layer and introduction of surface terminals in Ti_3_C_2_T_x_ MXene [48]. Figure 2b presents the FT–IR spectrum of the MXene. The characteristic absorption peaks of MXene at 3443, 1637, and 555 cm^−1^ correspond to the stretching vibrations of –OH, C=O, and Ti–O, respectively. The existence of functional groups such as –OH and –O on the surface of MXene is conducive to its further modification [49, 50]. These results confirmed the successful formation of Ti_3_C_2_T_x_ MXene sheets. The resulting multilayer Ti_3_C_2_T_x_ exhibited an accordion-like structure (Fig. S2b') and layered Ti_3_C_2_T_x_ MXene single and few-layer nanosheets obtained by ultrasonic treatment (Fig. S2b''). The FT–IR spectra of Pva-co-PE and PM_x_Ag are presented in Figs. S7 and 2c, respectively. The vibration peaks of the carbonyl group (–C=O) and stretching vibrations of –OH occurred at the 1641 and 3436 cm^−1^ bands, respectively (Fig. 2c). Comparing the FTIR spectra reveals that the absorption peak of the –OH group of the PM_x_Ag nanofiber membrane shifted to the right compared to that of Pva-co-PE, which proved the formation of hydrogen bonds between Pva-co-PE, MXene, and AgNW. Figures 2d–f and S8 show the XPS wide-scan spectra of the Pva-co-PE, Pva-co-PE/AgNW, and Pva-co-PE/AgNW/MXene nanofiber membranes, as well as the high-resolution spectra of C 1*s*, O 1*s*, and Ti 2*p* of the PM_x_Ag nanofiber membranes. As expected, the addition of MXene and AgNW introduced various polar groups, including oxides (–O–), fluorine (–F), and hydroxyl (–OH); characteristic peaks corresponding to N and Ag were observed in the corresponding spectra. In the high-resolution spectra of C 1*s* (Fig. 2e), there are apparent subpeaks corresponding to C–Ti, C–C, C–O, and C=O at 281.7, 284.8, 286.2, and 287.7 eV, respectively. The presence of C–O indicates that the MXene and Pva-co-PE nanofibers have numerous oxygen-containing functional groups. In addition, the carbon base (C=O) characteristic peaks of Pva-co-PE and PVP on the AgNW were observed in the C 1*s* spectra of the PM_x_Ag nanofiber membrane. Notably, compared to the Pva-co-PE nanofiber membrane (Fig. S8), the characteristic peaks of the PM_x_Ag composite membrane (C=O) shifted to a higher binding energy (287.7 eV). The results indicate that the chemical environment of –OH on the Ti_3_C_2_T_x_ MXene surface, Pva-co-PE surface, and C=O on the PVP surface of the AgNW changed, proving the existence of hydrogen bond interactions between MXene, Pva-co-PE, and AgNW. This is consistent with the results of the FTIR spectra. Numerous hydrogen bond interactions help enhance the interfacial force between MXene, Pva-co-PE, and AgNW, thereby improving the mechanical properties of the thin membranes [51]. The appearance of C-Ti was attributed to MXene, the structure of which was proven to be undamaged during the process of extraction and filtration while preparing the membrane. In the high-resolution spectrum of O 1*s* (Fig. 2f), there were apparent sub-peaks corresponding to O–Ti, C–Ti–O, C=O, C–O, and O–H at 529.3, 530.1, 532.5, 533.7, and 534.2 eV, respectively, which confirmed that the surface of MXene was rich in oxygen-containing functional groups. The aforementioned results demonstrated that the PM_x_Ag nanofiber composite membrane was successfully prepared and had a good interface force between the three components.

### Microstructure of the PM_x_Ag Nanofiber Composite Membrane

**Fig. 3:**
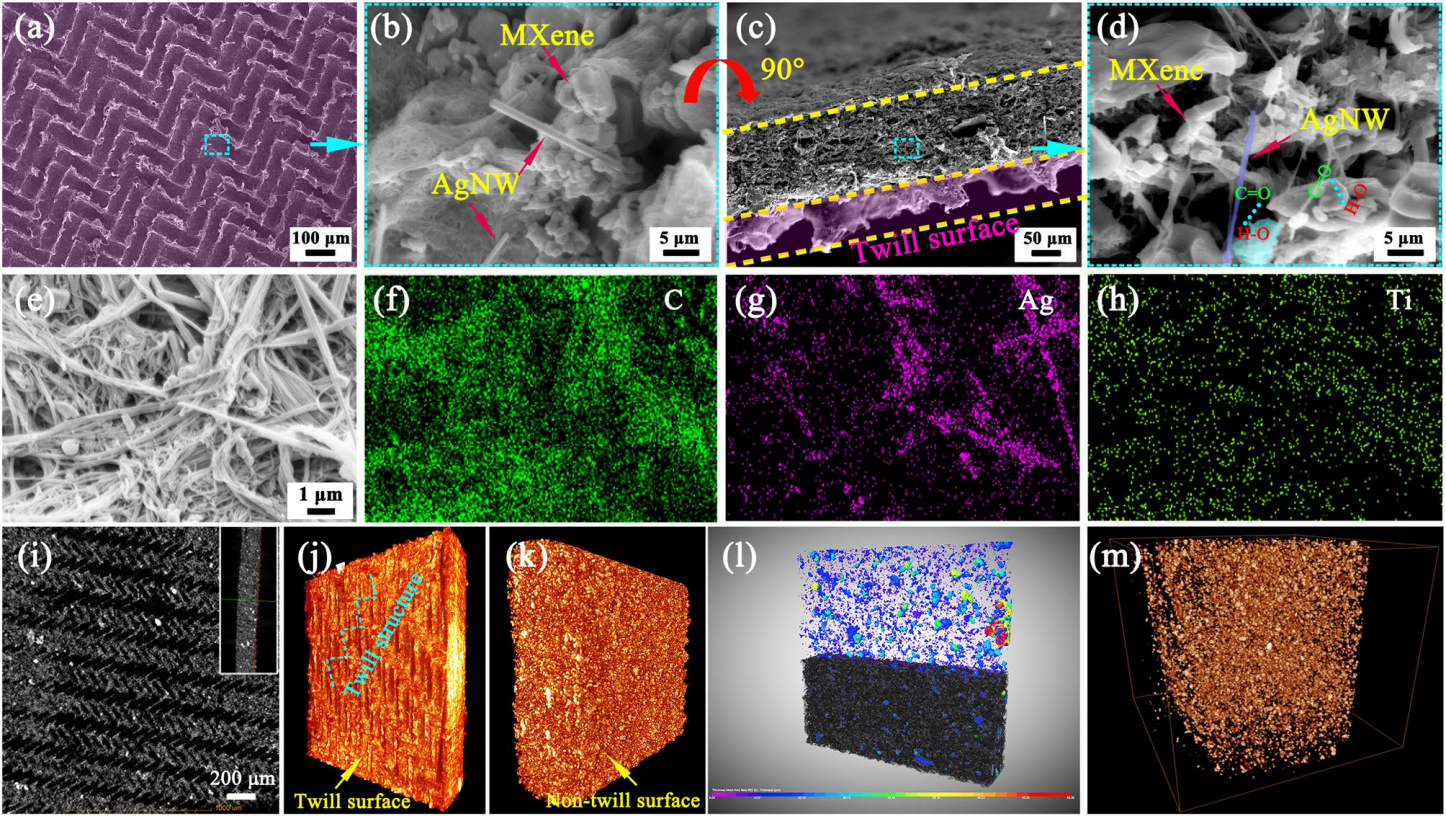
**a, b** Low- and high-magnification SEM images of the twill surface morphology of the PM_2.2_Ag composite membrane. **c, d** Low- and high-magnification SEM images of the cross-sectional morphology of the PM_2.2_Ag composite membrane. EDS elements on the surface of the PM_x_Ag nanofiber composite membrane, **e** morphology, **f** C, **g** Ag, and **h** Ti. **i** 3D–CT images**(inset:** 2D surface morphology). **j, k** 3D rendering of the membrane from two perspectives. **l** Dragonfly split of the 3D rendering diagram. **m** 3D rendering of the MXene/AgNW distribution in the composite membrane

Figure 3 presents a rendering diagram of the surface, cross section, elemental distribution, and 3D distribution of the absorbing media of the PM_x_Ag nanofiber composite membranes. Figure 3a–d shows the surface and cross-sectional morphologies of the PM_x_Ag nanofiber composite membranes. Figure S9 presents the cross-sectional SEM images of the different PM_x_Ag nanofiber composite membranes. A regular twill structure on the surface of the composite membranes and the shape of the cross-sectional twill structure are clearly observed (Figs. 3a and S9). Simultaneously, a uniform distribution of MXene and AgNW was observed in the Pva-co-PE nanofibers (Figs. 3d and S9). The formation of different thicknesses is mainly attributed to the fact that the introduction of MXene/AgNW increases the density of the nanofiber composite membranes, and the membrane thickness increases with the increase in dielectric mass, but is generally smaller than the thickness of the original membrane. Moreover, when comparing the electromagnetic shielding performance, we normalized the thickness. Figure 3e–h presents the distributions of the different elements, and the EDS spectra of PM_2.2_Ag (Fig. S10). The MXene/AgNW fillers were uniformly dispersed in the composite membranes. The surface morphology of the composite membrane and the 3D distribution of the filling medium in the membrane were observed (Fig. 3i–k) using an advanced 3D X-ray microscope (Xradia 515 Verse, Zeiss), and the results were consistent with the SEM results. The difference in density between the various components in the composite membrane was used to achieve the segmentation of different Pva-co-PE nanofibers, and a 3D rendering diagram of the distribution of the filling medium was constructed (Fig. 3l, m). The MXene/AgNW filling was uniformly distributed in the membrane, further proving that the excellent EMI SE was partly due to the uniform distribution of the absorbing medium in the membrane and the enhancement of various forms of the internal conduction loss of the EM waves.

### EMI Property of Nanofiber Composite Membranes

**Fig. 4:**
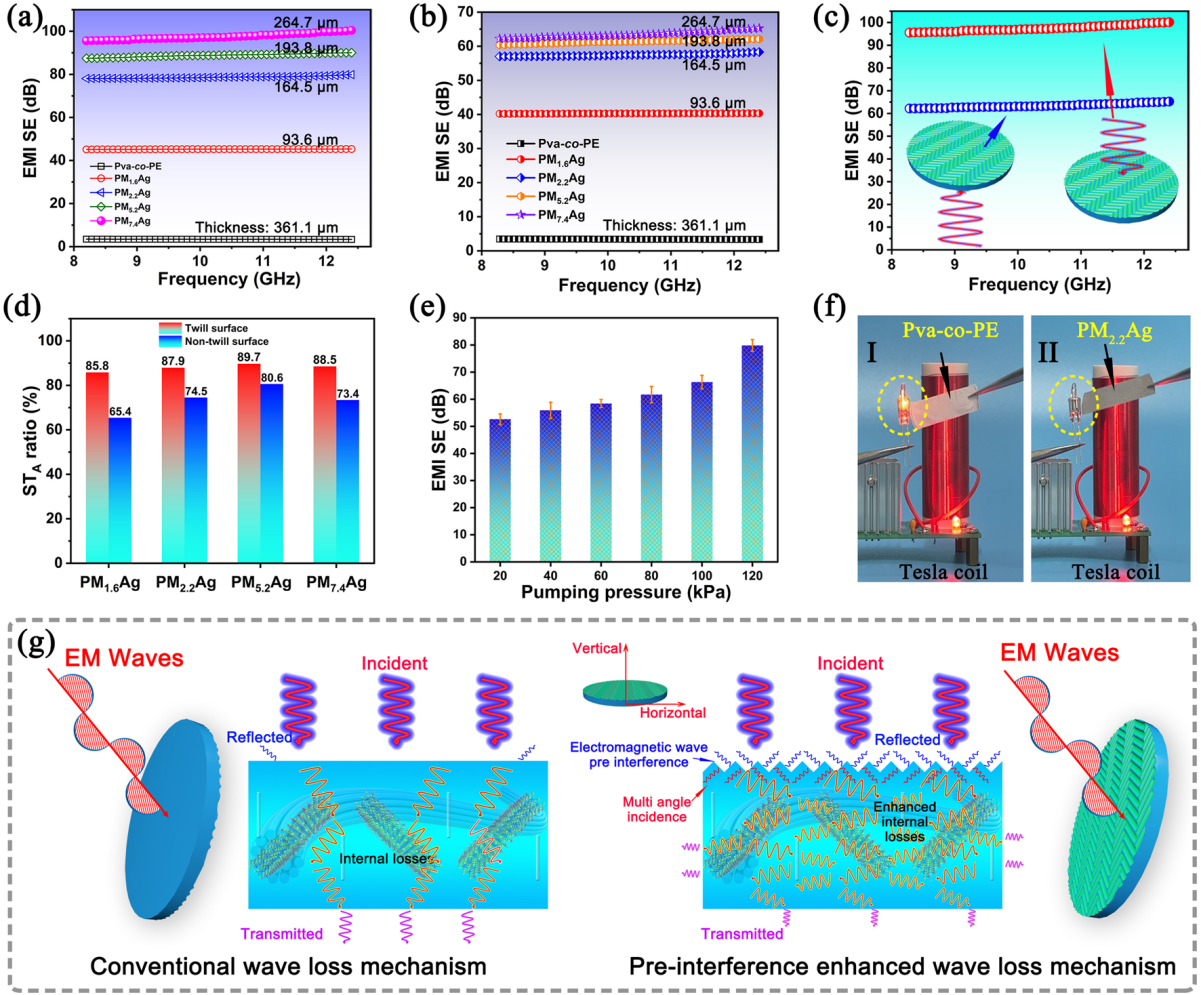
**a** EMI SE of the EM wave incident from the twill structure surface of the membrane. **b** EMI SE of the EM wave incident from the non-twill surface of the nanofiber membrane. **c** EMI SE of the PM_7.4_Ag nanofiber composite membrane after the EM wave was incident from two sides. **d** Comparison of the absorption ratios of the EM wave at different incidence planes. **e** EMI SE of the PM_2.2_Ag membranes with varying depths of the twill structure. **f** Physical demonstration of the modified PM_2.2_Ag membrane on a Tesla coil device. **g** EMI shielding mechanism diagram of EM wave incident on non-twill and twill structure surfaces

Figure 4a–c presents the EMI SE of nanofiber composite membranes that steadily fluctuatein the frequency range of 8.2–12.4 GHz (X-band). Figure 4a, b presents the EMI SE of the composite membranes against the EM waves incident from the twill and non-twill surfaces, respectively. The EMI SE of the composite membranes significantly improved as the MXene/AgNW content increased and the composite membranes showed better EMI SE when EM wave incident from the twill surface, therefore, we speculated that the surface twill structure had a positive effect on the electromagnetic interference shielding performance. Figure 4c presents a comparison of the EMI SE of the PM_7.4_Ag composite membrane when the EM wave entered from two different surfaces. The pre-interference twill structure clearly enhanced the EM wave transmission loss by 38.5%. To clarify the shielding mechanism of the composite membrane with twill surface, the contribution values of the total EMI shielding efficiency (SE_T_), wave reflection (SE_R_) and wave absorption (SE_A_) calculated by Eqs. S4 and S5 are shown in Fig. S11 and 4d. Figure S11 presents the EMI SE of composite membranes when the EM wave incident from different surfaces. The EMI SE of the composite membrane was significantly improved as the MXene/AgNW content increased, and the EM waves incident from the twill surface exhibited a better wave attenuation performance. The presence of the twill structure significantly improved the EMI SE of the membranes compared to that the EM waves entered from the non-twill surface. Figure 4d presents the SE_A_ of the PM_x_Ag composite membranes when EM waves are incident from different surfaces. The shielding properties of all the PM_x_Ag composite membranes demonstrated a higher SE_A_ contribution ratio (89.7%) when the EM waves were incident from the twill surface. With the increase of MXene/AgNW content, the SE_A_ contribution ratios of the composite membrane showed a significant upward trend when the EM wave was incident from the twill surface; however, the SE_R_ contribution ratio did not change significantly. Therefore, it can be judged that the substantial increase in EMI SE was mainly attributed to the increase in the contribution value of SE_A_. In addition, for the same composite membrane, the SE_A_ contribution ratio of EM wave incident from the twill surface was much higher than that of EM wave incident from the non-twill surface, and the SE_R_ contribution ratio hardly changed, as shown in Figs. 4d and S11j–l. The highly efficient conductive network constructed by MXene and AgNW, as well as the internal porous structure, enable the PM_x_Ag composite membrane to exhibit enhanced EM wave loss, which is attributed to energy dissipation and its conversion to heat. The pre-interference mechanism of the twill structure may increase the radial energy dissipation of more EM waves along the thin membrane (Fig. 4g).

To verify the influence of the surface of the twill structure on the EMI SE, we constructed twill structures of different depths on the PM_2.2_Ag composite membranes by controlling the pumping pressure; the surface SEM morphologies are shown in Fig. S12. The surface of the twill-structured nanofiber composite membrane was flexibly controlled by varying the suction pumping pressure, and the corresponding EMI SE values are shown in Fig. 4e. As the pumping pressure increased, the EMI SE of the EM waves increased significantly, verifying that the surface of the twill structure had a positive effect on the absorption performance of the EM waves. When the pumping pressure was high, the twill structure was more solid and the pre-interference efficiency in the direction of the incident EM wave was higher. However, when the pumping pressure was low, the surface twill structure profile was not obvious; thus, the pre-interference efficiency of the incident wave was poor. The EMI performance of the prepared composite membranes was directly verified using a Tesla EM induction coil system, whose circuit diagram is shown in Fig. S13. When the power supply is switched on, an electromagnetic field is first generated around the coil, and the potential generated by electromagnetic induction is then transmitted to the light-emitting diode, which immediately lights up. However, when the PM_2.2_Ag composite membrane was near the coil, the LED extinguished (Fig. 4f), which is attributed to the PM_2.2_Ag composite membrane cutting off the EM field around the coil (Movie S1). This result confirmed the effectiveness of the prepared PM_x_Ag composite membranes as high-performance EMI materials. In summary, the PM_x_Ag nanofiber composite membrane prepared in this study exhibited excellent EMI performance. Compared to other studies, the filling amount of MXene/AgNW in the PM_x_Ag composite membrane was small; therefore, its excellent EMI performance was closely related to the surface of the twill structure. The enhanced absorption mechanism is illustrated in Fig. 4g. The incident wave loss of the non-twill structure was mainly attributed to absorption caused by surface reflection and internal multistage reflection (left part in Fig. 4g). The incident wave loss of the twill structure was not only due to the enhanced wave reflection on the twill surface but also due to the change in the direction of the incident wave on the surface, which entered the nanofiber composite membrane in a multi-angle direction, inevitably increasing the reflection loss times of the EM waves inside the membrane (right part in Fig. 4g). The change in the incident direction of the EM wave was attributed to the high- and low-gradient distributions of the MXene/AgNW medium on the surface of the membrane, which caused the incident wave to change at different angles. This increased the probability of contact between the incident EM wave and MXene/AgNW inside the membrane, thereby increasing the wave loss. In addition, certain EM waves may transfer translationally along the membrane, resulting in a significant increase in the conduction path and wave loss.

**Fig. 5:**
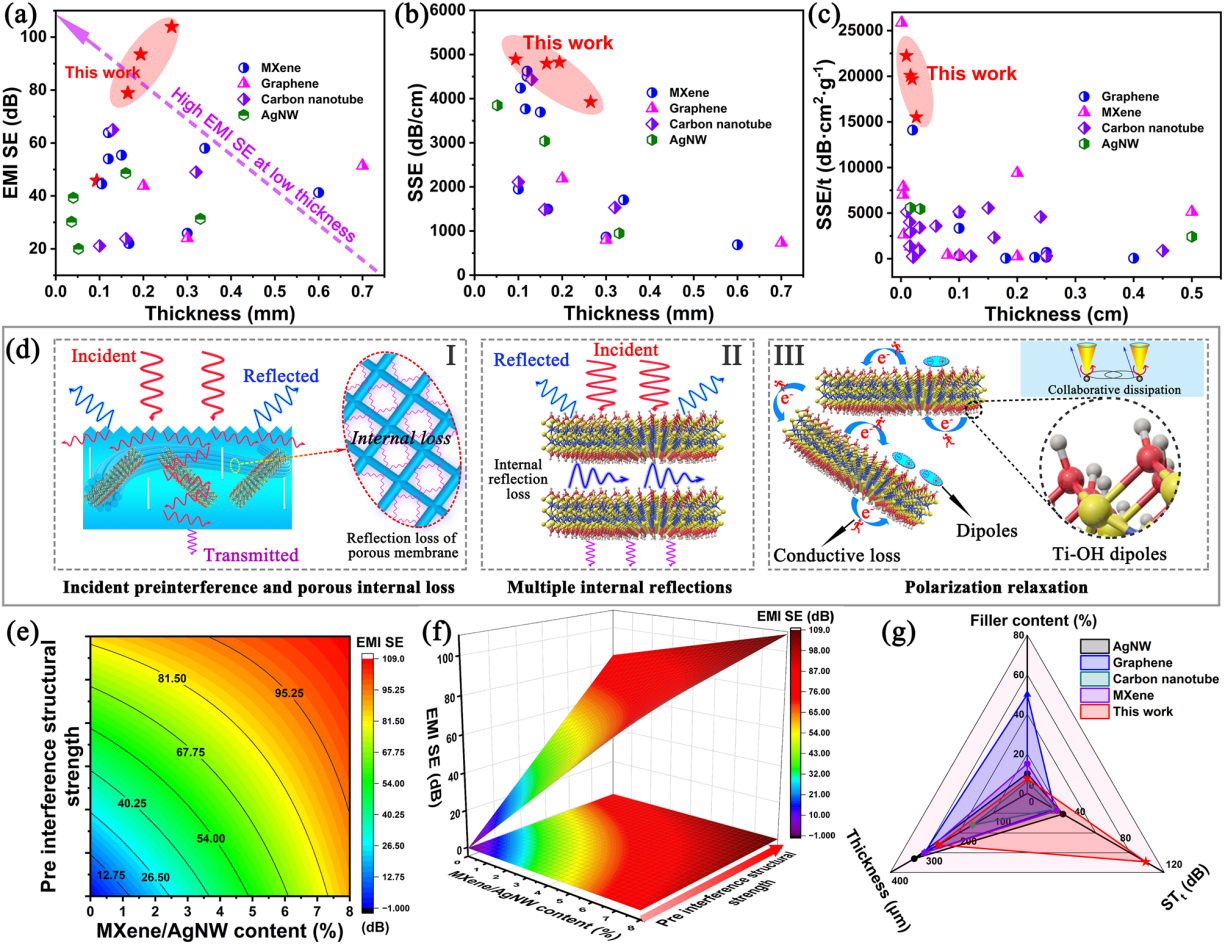
**a** PM_x_Ag composite membranes compared with the reported EMI materials. **b** EMI SE *versus* the thickness and **c** SSE versus the thickness of the PM_x_Ag composite membranes compared with previous studies. **d** EMI mechanism of the PM_x_Ag nanofiber composite membranes. **e** Contour map and **f** surface response model of the MXene/AgNW content and twill structure strength regarding the EMI SE. **g** Compared with the reported EMI materials [53–56], the PM_x_Ag nanofiber composite membranes demonstrated an excellent EMI SE

In addition to the effectiveness of EMI shielding, the thickness and weight were also considered as important criteria for evaluating high-performance EMI shielding materials, particularly those used in aerospace, military, artificial intelligence, smart wearable electronics, and apparels [29, 52]. Considering the influence of the thickness on the EMI SE, the performance was normalized by EMI SE/t to eliminate the influence of the thickness on the material properties. The normalization treatment (SSE/t) was critical for determining the EMI shielding performance of nanofiber membranes. Compared with the EMI SE, EMI SE/t, SSE/t, and the thickness, the advantages of the nanofiber composite membrane with the twill structure are highlighted compared with other shielding materials. As shown in Fig. 5a–c and Table S1, PM_x_Ag composite membranes with a twill structure simultaneously exhibited higher EMI SE, EMI SE/t, and SSE/t at ultra-low thicknesses and were ranked at the top of the comparison chart. In this study, a significantly low MXene/AgNW content of 2.2 wt% (164.5 μm) demonstrated a high EMI SE of 78.9 dB, EMI SE/t of 4824.6 dB cm^−1^, and SSE/t of 20,068.2 dB cm^2^ g^−1^, and other MXene/AgNW contents also demonstrated an excellent EMI performance, which was better than other organic nanofiber reinforced nanocomposite membranes. This was mainly due to the special twill structure that enabled the EM waves to enter the interior of the composite membrane at multiple angles, and the porous structure of the nanofiber membrane further enhanced the internal loss and absorption. The PM_x_Ag membrane obtained in this study achieved the multi-stage synergistic interference and absorption effects by the surface structure, absorbing medium, and internal porous structure of the membrane, significantly improving the EMI SE. This study provided a new strategy for preparing an electromagnetic shielding thin composite membrane with a high performance.

The EM wave transmission process of the PM_x_Ag nanofiber composite membrane used in this study is illustrated in Fig. 5d. When EM waves impact the surface of a composite membrane with a twill structure, certain incident EM waves reach the MXene/AgNW surface and are immediately reflected owing to the impedance mismatch. In addition, the formation of a gradient twill structure causes the direction of the EM waves to change to different degrees and enter the membrane (Fig. 5d–I). Multiple angles of incident EM waves increased the number of wave reflection losses as well as the impact probability of EM waves and MXene/AgNW. Moreover, when the incident EM wave passes through the conductive MXene/AgNW layer at multiple angles, it interacts with high-density electron carriers (such as electrons, holes, and dipoles), resulting in the attenuation of the EM wave energy through the induction current, combined with the natural resonance loss between the electromagnetic waves and the medium, and finally intensely absorbs the electromagnetic wave (Fig. 5d–II, III). In addition, the special porous structure of the nanofiber membrane further increased the reflection loss of the electromagnetic waves (Fig. 5d–I). Moreover, multiple internal reflections between adjacent MXene nanosheets promoted the dissipation and attenuation of electromagnetic waves, which were eventually fully absorbed (Fig. 5d–II). Meanwhile, after stripping, Ti_3_C_2_T_x_ MXene offers numerous end groups and can induce an asymmetric charge density distribution at the surface, edge, and other local dipoles, resulting in a directional rotation toward the electromagnetic field, polarization relaxation, and enhancement of the overall shielding effect (Fig. 5d–III). For the PM_x_Ag membranes, the EMI performance was mainly ascribed to the pre-interference of the surface twill structure on the direction of the EM wave and the enhanced electromagnetic reflection and absorption mechanisms indicated above. This excellent EMI performance proved that the surface twill structure has an important influence on the enhanced shielding performance. To better study the relationship among the surface twill structure, MXene/AgNW content, and EMI SE, we built an optimization model (the theoretical equation follows Eqs. S10 and S11), and the results are shown in Fig. 5e, f. The role of the twill structure on the EMI SE is apparently nearly equal to the importance of the MXene/AgNW content. For the same EMI SE, the surface twill structure introduced significantly reduced the filling content of the MXene/AgNW. The introduction of this new concept is practically significant for retaining the flexible characteristics of the nanofiber membrane matrix and reducing the cost of the MXene/AgNW filling. A comparison of similar electromagnetic shielding membranes is shown in Fig. 5g. The simple preparation process, low media filling capacity, and high electromagnetic shielding performance of the PM_x_Ag nanofiber composite membrane are useful in many fields such as smart wearable and flexible electronic products.

### Mechanical Properties of the PM_x_Ag Composite Membranes

**Fig. 6:**
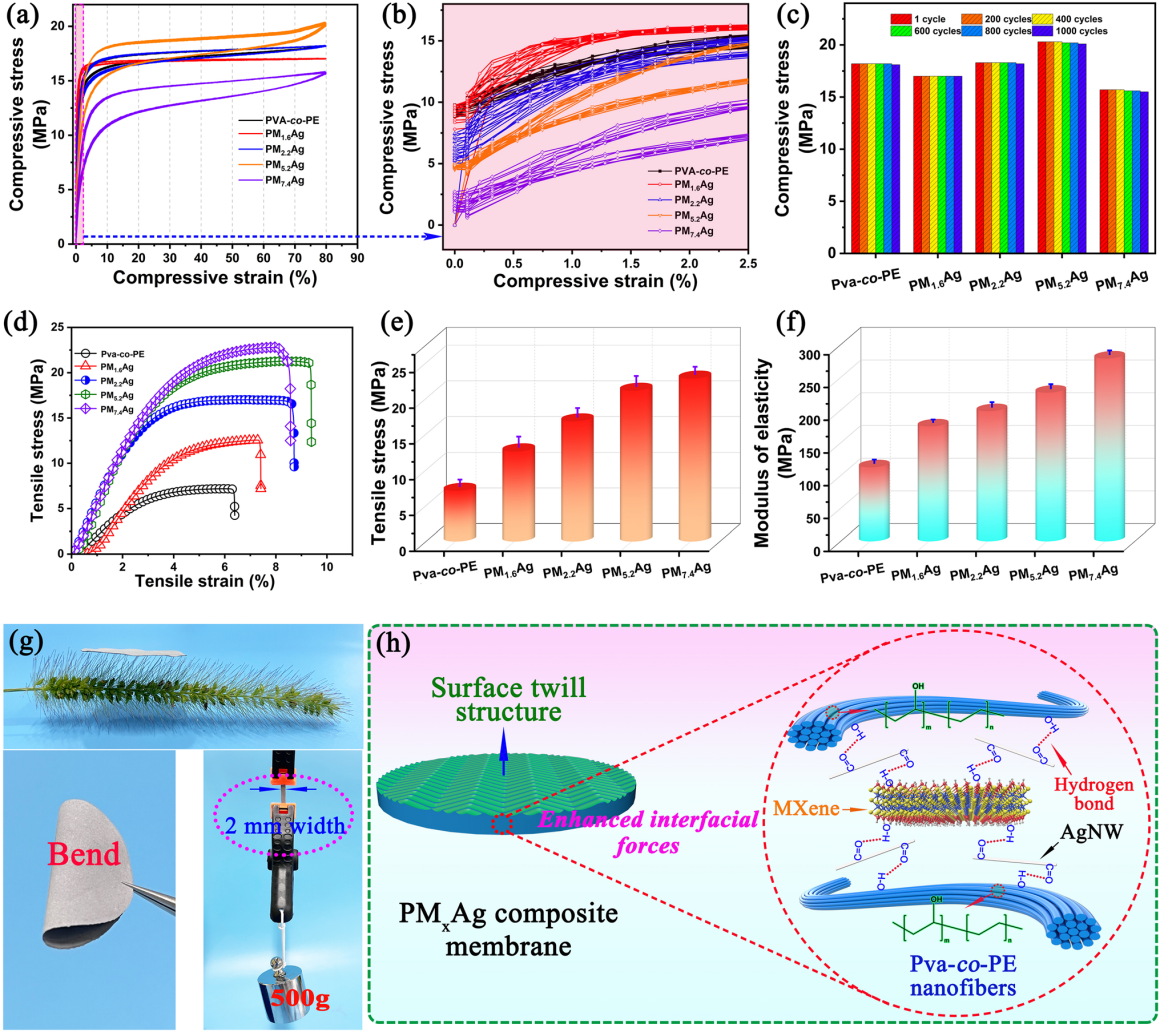
**a, b** Low- and high-magnification cyclic compression curves of the PM_x_Ag membranes for 100 cycles. **c** Stress values of various cycle compression times. **d** Tensile stress–strain curve, **e** tensile stress, and **f** elastic modulus of different PM_x_Ag composite membranes. **g** Digital images of the PM_x_Ag composite membrane that is ultra-light, ultra-tough, and can withstand a weight of 500 g. **h** Schematic diagram of the interaction mechanism between the three components of Pva-co-PE, MXene, and AgNW

In this study, a composite membrane with a high shielding property for EM waves prepared by using nanofibers as a matrix can be broadly applied in intelligent wearable, flexible electronic products, and other fields; therefore, its mechanical properties have become an important concern. Figure 6a, b presents the compressive stress–strain curves of the nanofiber composite membrane after 100 cycles. When the filling content of the MXene/AgNW was between 1.6 and 7.4 wt%, the compressive stresses did not change significantly, and the multiple cyclic compression curves nearly overlapped, indicating that the PM_x_Ag composite membranes possessed an excellent cyclic compression stability. Figure 6c demonstrates a statistical diagram of the stress after various cyclic compression cycles. After 1000 cycles of cyclic compression, the various PM_x_Ag nanofiber composite membranes remained to demonstrate an excellent cyclic compression stability, where the compression stresses were maintained above 98%. A comparison with the Pva-co-PE nanofiber membrane also demonstrated that the filling of MXene/AgNW had a limited influence on the multi-cycle compression performance. The tensile properties of the nanofiber membranes with different components are plotted in Fig. 6d–f. As the MXene/AgNW content increased, the tensile strength of the nanofiber composite membrane increased (Fig. 6d), where the tensile stress and modulus significantly increased to 22.8 and 279.1 MPa (Fig. 6e, f), respectively. Figure 6g presents a digital image of the ultralight, ultraflexible, and high-tensile properties of the nanofiber composite membrane. Based on these results, the PM_x_Ag nanofiber composite membrane demonstrated excellent mechanical properties. The ultralight intensity was owing to the low filling content of the MXene/AgNW and the internal porous structure. This flexibility was ascribed to the unique characteristics of the ultrathin porous nanofiber membranes. The excellent tensile and cyclic compression properties were attributed to the forces between the components of the nanofiber composite membrane. The abundant –OH on the surface of the Pva-co-PE nanofibers and MXene nanosheets can easily react with AgNW containing –C=O to form O–H bonds, and the AgNW act as an intermediate medium to “lock” the three components to form a stable and high-strength composite structure (Fig. 6h). In addition, AgNW with a length-to-diameter ratio physically interact with the porous structure of the nanofiber membrane, further enhancing the mechanical properties of the composite membrane.

### Thermal Management Properties of the PM_x_Ag Composite Membranes

**Fig. 7:**
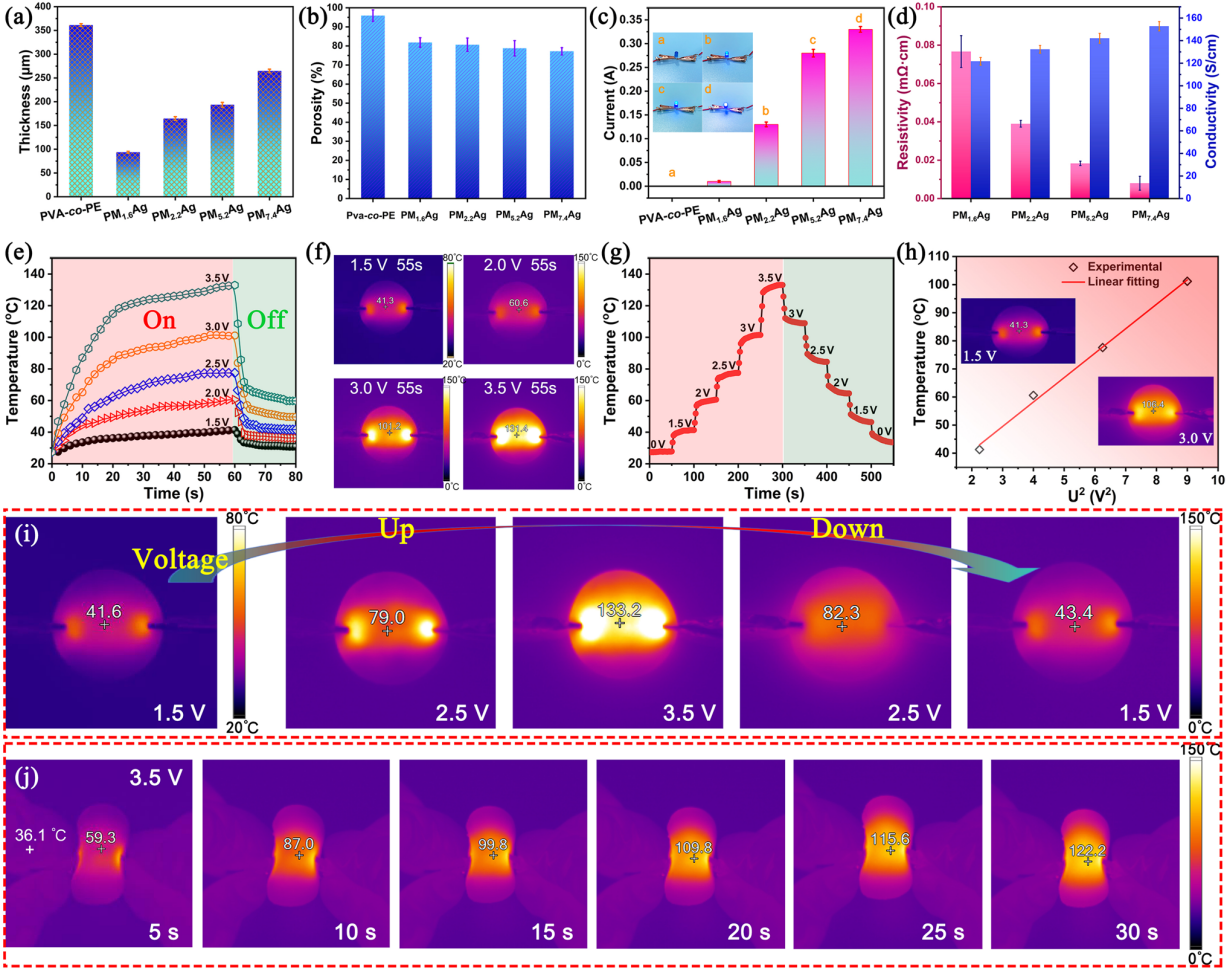
**a** Thicknesses and **b** porosities of different PM_x_Ag membranes. **c, d** Electrical conductivities. **e** Time-dependent surface temperatures of the PM_5.2_Ag membrane. **f** Surface temperatures of the PM_5.2_Ag membrane at different voltages at 55 s. **g** Tailored surface temperatures of the PM_5.2_Ag membrane. **h** Experimental data and linear fitting of the saturation temperature *versus* U^2^. **i** Fluke IR camera images of the tailored surface temperatures of the PM_5.2_Ag membrane. **j** Real-time infrared image of the rapid surface heating of the PM_7.4_Ag membrane

In addition to the excellent mechanical properties and electromagnetic shielding performance of the PM_x_Ag nanofiber composite membrane with a twill structure, it can also be used as a high-performance Joule heater, demonstrating significant potential in emerging applications such as smart fabrics as well as wearable electronic fabrics. Figure 7a presents the thickness statistics of the nanofiber composite membranes with different amounts of MXene/AgNW filling. The thickness of the original Pva-co-PE membrane was 361.1 μm. When the amount of MXene/AgNW was 1.6 wt%, the membrane thickness decreased to 93.6 μm, and subsequently gradually increased as the MXene/AgNW content increased. This can be attributed to the fact that the MXene/AgNW filling increased the density of the nanofiber composite membranes, and the increased amounts further promoted the accumulation of MXene and AgNW, thereby enhancing the interfacial interactions between MXene, AgNW, and nanofibers. This promoted the formation of a perfect thermal and conductive network inside the nanofiber composite membrane. The porosities of the PM_x_Ag composite membranes are shown in Fig. 7b. All the composite membranes maintained a porosity of greater than 70%, which was attributed to the low MXene/AgNW content and the porous structure of the nanofiber membrane. Figure 7c, d presents the electrical conductivities of the PM_x_Ag nanofiber composite membranes. As the MXene/AgNW content increased, a more efficient MXene/AgNW conductive network was formed inside the membrane, where the conductivity was improved. For example, when the MXene/AgNW content increased from 0 to 7.4 wt%, the conductivities increased from 0 to 152.3 S cm^−1^ (Fig. 7d). The low content of the MXene/AgNW and higher electrical conductivity indicate that the MXene/AgNW improve the uniformity of the MXene/AgNW conductive network along the nanofiber distribution, and MXene/AgNW also entered the nanofiber holes to further reinforce the conductive network. Figure S14 presents an SEM image of the morphology of the PM_7.4_Ag nanofiber composite membrane after combustion, which clearly demonstrates that the MXene/AgNW exhibited a good uniformity of distribution. Moreover, the MXene thin sheets and AgNW exhibited a good interfacial combination, which further verified the good conductive network inside the nanofiber composite membrane.

Figure 7e presents the time-dependent surface temperature of the PM_5.2_Ag nanofiber composite membrane (radius = 1.5 cm). At a low power supply voltage, the ultra-flexible nanofiber composite membrane heats up from room temperature (25.3 °C) to 41.3 °C (55 s), reaching an appropriate temperature for wearable thermotherapy to remain warm or alleviate pain and stiffness. As the power supply voltage increased, the steady-state saturation temperature significantly increased owing to the larger Joule heat generated by the higher passing current; furthermore, when the power supply was stopped, all the composite membranes rapidly dissipated the heat within 10 s (Fig. 7e, f). As shown in Fig. 7g, the surface temperature of the nanofiber composite membrane was easily adjusted by changing the power-supply voltage owing to the highly responsive Joule heat generated by the PM_x_Ag composite membrane. Figure 7i presents a real-time image of the composite membrane surface temperature varying with the power supply voltage. Figure 7h presents the experimental data, linear fitting of the surface saturation temperature, and U^2^. Notably, the surface temperature of the nanofiber composite membrane was linearly related to the square of the power supply voltage, which is consistent with the theoretical prediction of electric heaters reported in previous studies [29, 51, 57, 58]. Figure 7j presents the rapid surface temperature response of the PM_7.4_Ag composite membrane under bending at a supply voltage of 3.5 V, which was owing to the formation of a good and stable conductive and thermal network inside the membrane by the Pva-co-PE, MXene, and AgNW. These results demonstrate that the composite membrane can achieve rapid low-voltage heating and heat dissipation, and has potential applications in the thermal management of flexible intelligent electronic products.

### Hydrophilic, Flame Retardant, and Performance Stability of the Nanofiber Composite Membrane

**Fig. 8:**
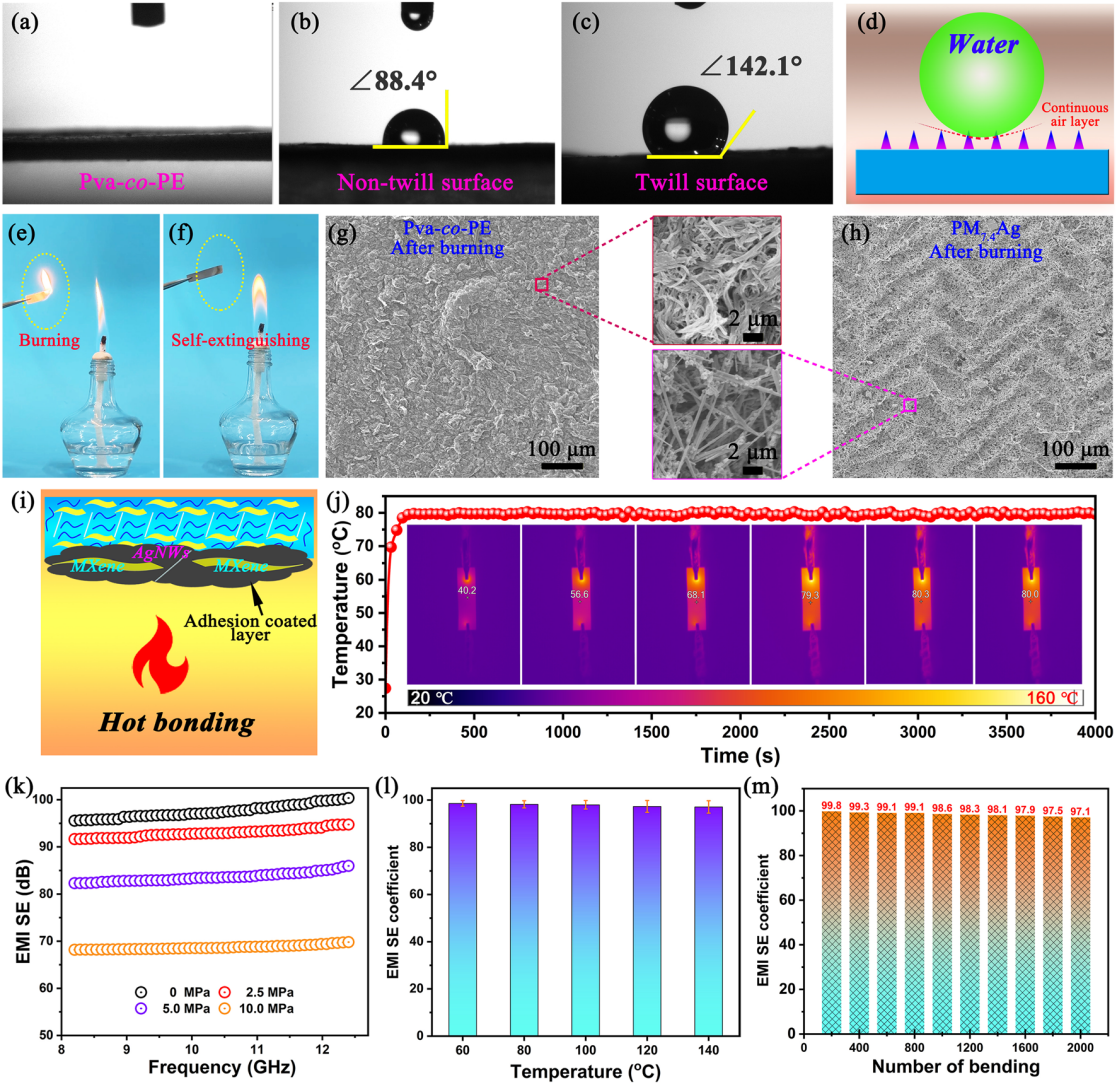
**a–c** Water contact angles of the Pva-co-PE and PM_7.4_Ag membranes. **d** Mechanism of the hydrophobicity improvement of the PM_x_Ag composite membranes. **e, f** Digital photographs of the flame-retardant testing of the Pva-co-PE and PM_7.4_Ag membranes. **g, h** SEM images of Pva-co-PE and PM_7.4_Ag after combustion. **i** Self-extinguishing combustion mechanism of the PM_5.2_Ag composite membrane. **j** Long-term time–temperature curve at a constant voltage of 2.5 V for the PM_5.2_Ag nanofiber composite membrane. **k** EMI SE of the PM_7.4_Ag composite membrane subjected to different compressive stresses on twill surfaces. **l, m** EMI efficiency of the PM_7.4_Ag nanofiber composite membrane at different ambient temperatures and different number of bending, respectively

To adapt to complex and varying application environments, high-performance EM wave absorption materials often need to possess various properties such as hydrophobicity, high-temperature resistance, nonflammability, and performance stability. Excellent hydrophobicity can effectively protect intelligent wearable electronic devices from damage caused by moisture. The original Pva-co-PE nanofiber membrane is hydrophilic (Fig. 8a). However, the PM_x_Ag nanofiber composite membranes exhibited excellent hydrophobicity with a water contact angle of 142.1°, as shown in Fig. 8c and Movies S2, S3. The water contact angle of the non-twill surface of the PM_7.4_Ag composite membrane is 88.4° (Fig. 8b). This was mainly due to the twill structure on the surface of the nanofiber composite membrane and the presence of the MXene/AgNW media significantly increasing the surface roughness, whereas the regular twill structure of the surface promoted the simple formation of a continuous air layer between the water droplets and the surface (Fig. 8d), providing the nanofiber composite membrane with an excellent hydrophobicity. Figure S15 shows the change in the water contact angle on the twill surface of the composite membrane after different bending times. The composite membrane exhibited excellent hydrophobicity and surface structural stability.

When exposed to the environment, the excellent hydrophobicity of the composite membrane renders it waterproof, frost resistant, and corrosion resistant. The combustibility and self-quenching performance of PM_x_Ag were evaluated using air combustion tests. As shown in Fig. 8e, f, compared to the Pva-co-PE membrane, after the PM_x_Ag nanofiber composite membrane contacted the open flame, the Pva-co-PE fibers rapidly adhered to the MXene/AgNW medium to form a dense incomplete combustion coke layer, which delayed combustion without melting the droplets or self-quenching the flame (Fig. 8i). Figure 8g, h presents the SEM images of the combustion interface morphologies of the Pva-co-PE and PM_7.4_Ag nanofiber membranes, respectively. The combustion interface of the Pva-co-PE nanofiber membrane consisted of unburned nanofibers, whereas the combustion interface of PM_7.4_Ag consisted of an MXene/AgNW complex and coke particles of incomplete nanofibers, forming a flame-retardant protective layer that can promote rapid heat dissipation during combustion (Movies S4 and S5). Figure 8j shows the heating properties of long-term PM_5.2_Ag nanocomposite membranes. As can be seen, the nanocomposite membrane presents a very stable surface temperature of approximately 80 °C for a duration of more than 1 h, demonstrating its excellent Joule thermal stability and reliability for thermal management applications. Figure S16 shows the changes in the surface morphology and water contact angle of the twill structure on the surface of the PM_7.4_Ag composite membrane subjected to different compressive stresses. When subjected to large compressive stress, the surface twill structure can still show a certain twill structure, especially when subjected to 10 MPa compressive stress, while the water contact angle and EMI SE are still higher than 95° and 66 dB (Figs. S16c'' and 8k, respectively). Subsequently, the stability of the PM_x_Ag nanofiber membrane was studied considering the EMI performance at higher temperatures and multiple bending cycles, as shown in Fig. 8l, m, which demonstrate that the shielding efficiency of the PM_5.2_Ag electromagnetic waves remained above 97% at high ambient temperatures. For example, when the ambient temperature was 140 °C, the EMI SE coefficient was 97.3%. Moreover, after 2000 cycles of bending, the shielding efficiency was 97.1%. The aforementioned EMI SE is attributed to the stable skeleton structure inside the PM_x_Ag nanofiber membrane, as shown in Fig. S17. The physicochemical interactions between Pva-co-PE, MXene, and the AgNW significantly improved the interfacial forces. Combined with the inherent flexibility of the Pva-co-PE nanofibers, excellent flexibility and tensile properties were demonstrated, thereby ensuring the stability of their outstanding properties. In summary, the PM_x_Ag nanofiber membrane with a twill structure demonstrated excellent and stable high-performance electromagnetic shielding, hydrophobicity, and noncombustible properties.

## Conclusion

This study successfully demonstrated the preparation of a flexible PM_x_Ag composite membrane with a gradient twill structure. A combination of vacuum filtration and a template method was used to achieve a low MXene/AgNW filling capacity and high-performing EMI. The physicochemical interactions between the Pva-co-PE nanofibers, MXene, and AgNW synchronized the construction of an efficient three-dimensional conductive network and a stable three-dimensional structural system, which enabled the composite membrane to exhibit a high conductivity and electrical stability during repeated bending and stretching. These results demonstrate that the pre-interference of the surface twill structure on the direction of the electromagnetic wave incidence, the large ohmic loss of the conductive network, reflection loss between the MXene nanosheets, and enhancement of the reflection loss by the porous structure of the nanofiber membrane are the main reasons for the excellent electromagnetic shielding performance of the MXene nanosheets. When the amount of MXene/AgNW was only 7.4 wt%, the EMI SE of the nanofiber composite membrane with a twill structure on the surface was 103.9 dB, which benefitted from the pre-interference of the surface twill structure on the direction of the incident electromagnetic wave and effectively enhanced the probability of the disorderly collision between the electromagnetic waves and MXene nanosheet. The internal reflection, ohmic, and resonance losses of the membrane were simultaneously enhanced. The construction of a surface twill solves the problems of a low MXene/AgNW filling capacity and high electromagnetic shielding performance, which are difficult to achieve. The PM_7.4_Ag composite membrane with a surface twill structure exhibits both an outstanding tensile strength of 22.8 MPa and EMI SE/t of 3925.2 dB cm^−1^, which is attributed to the extensive hydrogen bonding between the Pva-co-PE, MXene, and AgNW. In addition, the PM_x_Ag nanofiber membrane demonstrated excellent thermal management properties, such as a low voltage and high Joule heat, rapid heating response, rapid heat dissipation, and an excellent stability. In addition, the PM_x_Ag composite membrane exhibited outstanding hydrophobicity, flame retardancy, and EMI SE stability in complex environments. For the first time, a surface twill structure was constructed on a nanofiber composite membrane, which achieved the simple and efficient preparation of a composite membrane with a low media-filling capacity and high electromagnetic shielding performance. Simultaneously, it exhibited excellent flexibility, a good thermal management performance, hydrophobic and flame-retardant properties, and a stable performance in complex environments. It has significant potential in the field of EMI shielding protection for smart portable fabrics and flexible wearable electronic devices.

## Supplementary Information

Below is the link to the electronic supplementary material.

Supplementary file1 (MP4 1520 KB)

Supplementary file2 (MP4 965 KB)

Supplementary file3 (MP4 756 KB)

Supplementary file4 (MP4 441 KB)

Supplementary file5 (MP4 930 KB)

Supplementary file6 (DOCX 32825 KB)
