# Supplementary material for: Controlled Twill Surface Structure Endowing Nanofiber Composite Membrane Excellent Electromagnetic Interference Shielding
Source: Nanomicro Lett. 2024 Jul 4;16:236. doi: 10.1007/s40820-024-01444-y (PMC11224063; doi:10.1007/s40820-024-01444-y)
Supplement: Supplementary file 6 — Supplementary file6 (DOCX 32825 KB) [file 40820_2024_1444_MOESM6_ESM.docx]

Supporting Information for

**Controlled Twill Surface Structure Endowing Nanofiber Composite Membrane Excellent Electromagnetic Interference Shielding**

Dechang Tao ^1, #^, Xin Wen ^1, 2, #^, Chenguang Yang^1,^ *, Kun Yan^1^, Zhiyao Li ^1^, Wenwen Wang^1,^ *, Dong Wang^1, 2,^ *

^1^ Key Laboratory of Textile Fiber and Products (Wuhan Textile University), Ministry of Education, Wuhan Textile University, Wuhan 430200, P. R. China

^2^ College of Chemistry, Chemical Engineering and Biotechnology, Donghua University, Shanghai 201620, P. R. China

# Dechang Tao and Xin Wen contributed equally to this work.

*Corresponding authors. E-mail: [cgyang@wtu.edu.cn](mailto:cgyang@wtu.edu.cn) (Chenguang Yang); [wwang@wtu.edu.cn](mailto:wwang@wtu.edu.cn) (Wenwen Wang); [wangdon08@126.com](mailto:wangdon08@126.com) (Dong Wang)

**S1 Experimental Section**

**S1.1 Preparation of nanofiber membrane**

Polyvinyl alcohol-polyethylene copolymer (Pva-co-PE) and cellulose acetate butyrate (CAB) are uniformly mixed in a mass ratio of 2:8, and then extruded using a twin-screw extruder with a screw diameter of 28 mm and a length-to-diameter ratio of 35 to prepare new-born island fibers. The precursor fibers are extruded using a spinneret with a diameter of 1.8 mm and 12 holes, and spun under the conditions of 200~220°C, 1.5~2.5 MPa, and a winding speed of 1.5 m/s. After removing the sea phase with acetone, nanofibers are obtained, and the process diagram for the preparation of nanofibers is shown in Fig. S1.


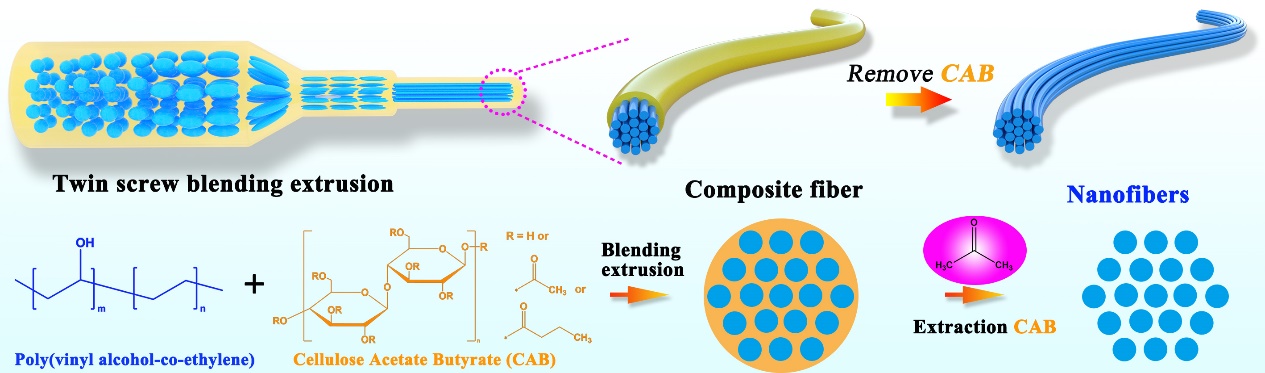


**Fig. S1** Schematic diagram of preparation of Pva-co-PE nanofibers

The nanofiber suspension membrane was prepared by using Pva-co-PE nanofibers with a diameter of 100-200 nm. The preparation process of nanofiber is shown in Fig. S1 and the SEM images of the nanofibers are shown in Fig. S2 c, c'. Ti_3_AlC_2_ (MAX) powder (98.7%) was obtained from Suzhou Kaifa New Material Technology Co., LTD. (Suzhou, China). Lithium fluoride (LiF), hydrochloric acid (HCl, 33wt.%), potassium hydroxide (KOH), silver nitrate (AgNO_3_), ethylene glycol (EG), copper chloride dihydrate (CuCl_2_·2H_2_O), ferric chloride (FeCl_3_), sodium bromide (NaBr) and polyvinylpyrrolidone (PVP, Mw=1300000 g/mol) were purchased from Sinopharm Chemical Reagent Co., Ltd. Nylon fabric (JRYS014, hole size: 25 μm×50 μm) provided by Suzhou Jurui textile strength Co., Ltd. The SEM images of the twill structure on the surface of nylon fabric are shown in Fig. S3.

**S1.2 Synthesis of Ti_3_C_2_T_x_ MXene**

Fig. S4 shows the preparation diagram of Ti_3_C_2_T_x_ MXene. First, 4g LiF was dissolved in a mixture of 60 mL HCl and 20 mL deionized water to make an etching solution. Under magnetic agitation, 2g Ti_3_AlC_2_ was added to the prepared etching solution and stirred at 35°C for 48 h. The mixture was centrifuged at 4000 r/min for 5 mins each time, and the product was repeatedly washed with deionized water until the pH was 6. Finally, the supernatant was collected by ultrasound for 30 min and centrifugation at a speed of 3500 r/min for 60 minutes, and a dark green colloidal solution of Ti_3_C_2_T_x_ MXene was obtained.

**S1.3 Synthesis of Silver Nanowires**

By magnetic stirring, 1.6g amount of PVP was fully dissolved in 100 mL EG in a three-necked flask at 150°C for 4 hours. Subsequently, 4 mL of 20 mM CuCl_2_·2H_2_O and 4 mM ferric chloride were added to PVP solution to achieve ultra-high aspect ratio AgNWs. A mixture of 40 mL of 0.70 M AgNO_3_ and 4 mM sodium bromide was then added to the solution and left for 2 hours at 150°C. After cooling to room temperature, filter acetone and ethanol with inhalation to wash completely. The obtained AgNWs were re-dispersed in deionized water at a concentration of 2.5 mg·mL^−1^ for future use. The optical photos of silver nanowire solution and microphotograph of the obtained AgNWs are shown in Fig. S2 a and a', respectively.

**S1.4 Electromagnetic shielding effectiveness: theory and measurement**

The EMI SE was analyzed using a vector network analyzer (Agilent, PNA-N5244A) in the frequency range of 8.2−12.4 GHz. Scattering parameters (S11 and S21) were recorded, and transmission (T), reflection (R), and absorption (A) coefficients were calculated. The total EMI SE (SE_T_) and contributions from reflection (SE_R_), absorption (SE_A_) and multiple internal reflections (SE_M_) are calculated as follows:

$\text{R=}\text{S}_{\text{11}}^{\text{2}}$ (S1)

$\text{T=}\text{S}_{\text{12}}^{\text{2}}\text{=}\text{S}_{\text{21}}^{\text{2}}$ (S2)

$\text{A=1-T-R}$ (S3)

$\text{S}\text{E}_{\text{R}}\left( \text{dB} \right)\text{=-10}\log\left( \text{1-}\text{S}_{\text{11}}^{\text{2}} \right)\text{=-10}\log\left( \text{1-R} \right)$ (S4)

$\text{S}\text{E}_{\text{A}}\left( \text{dB} \right)\text{=-10}\log\left( \frac{\text{S}_{\text{12}}^{\text{2}}}{\text{1-}\text{S}_{\text{11}}^{\text{2}}} \right)\text{=-10}\log\left( \frac{\text{T}}{\text{1-R}} \right)$ (S5)

$\text{S}\text{E}_{\text{T}}\left( \text{dB} \right)\text{=S}\text{E}_{\text{A}}\text{+S}\text{E}_{\text{R}}$ (S6)

EMI SE depends on the dielectric and magnetic properties. At the same time, the EMI SE/t is normalized to eliminate the influence of thickness. In addition, the specific shielding efficiency (SSE) and SSE/t, taking into account density and thickness, are expressed as follows:

$\text{SSE}\text{=}\text{EMI}\frac{\text{SE}}{\text{density}}\text{=}\text{dB}\text{∙}\text{c}\text{m}^{\text{3}}\text{∙}\text{g}^{\text{-1}}$ (S7)

$\text{SSE}\text{/}\text{t}\text{=SSE/thickness=}\text{dB}\text{∙}\text{c}\text{m}^{\text{2}}\text{∙}\text{g}^{\text{-1}}$ (S8)

EMI shielding efficiency (%) is obtained by using the following equation:

$\text{Shield efficiency (\%) =100-(}\frac{\text{1}}{\text{10}^{\text{SE}/\text{10}}}\text{)×100}$ (S9)

**S1.5 Optimization model design**

A typical square root optimization model (Eq. S10) is used in this work.

$y^{'}=y$ (S10)

The process sequence is executed according to the main effect. The optimal design software Design-Expert 12.0 was used to obtain the final equation (Eq. S11) according to the actual factors.

$$\text{EMI SE (dB) =-0.931216+11.24464*MXene/AgNW content (\%) +14.17228*pumping pressure}$$

$\text{ (kPa) -1.36029*MXene/AgNW content (\%)*pumping pressure (kPa)}$ (S11)

**S2 Supplementary Tables and Figures**


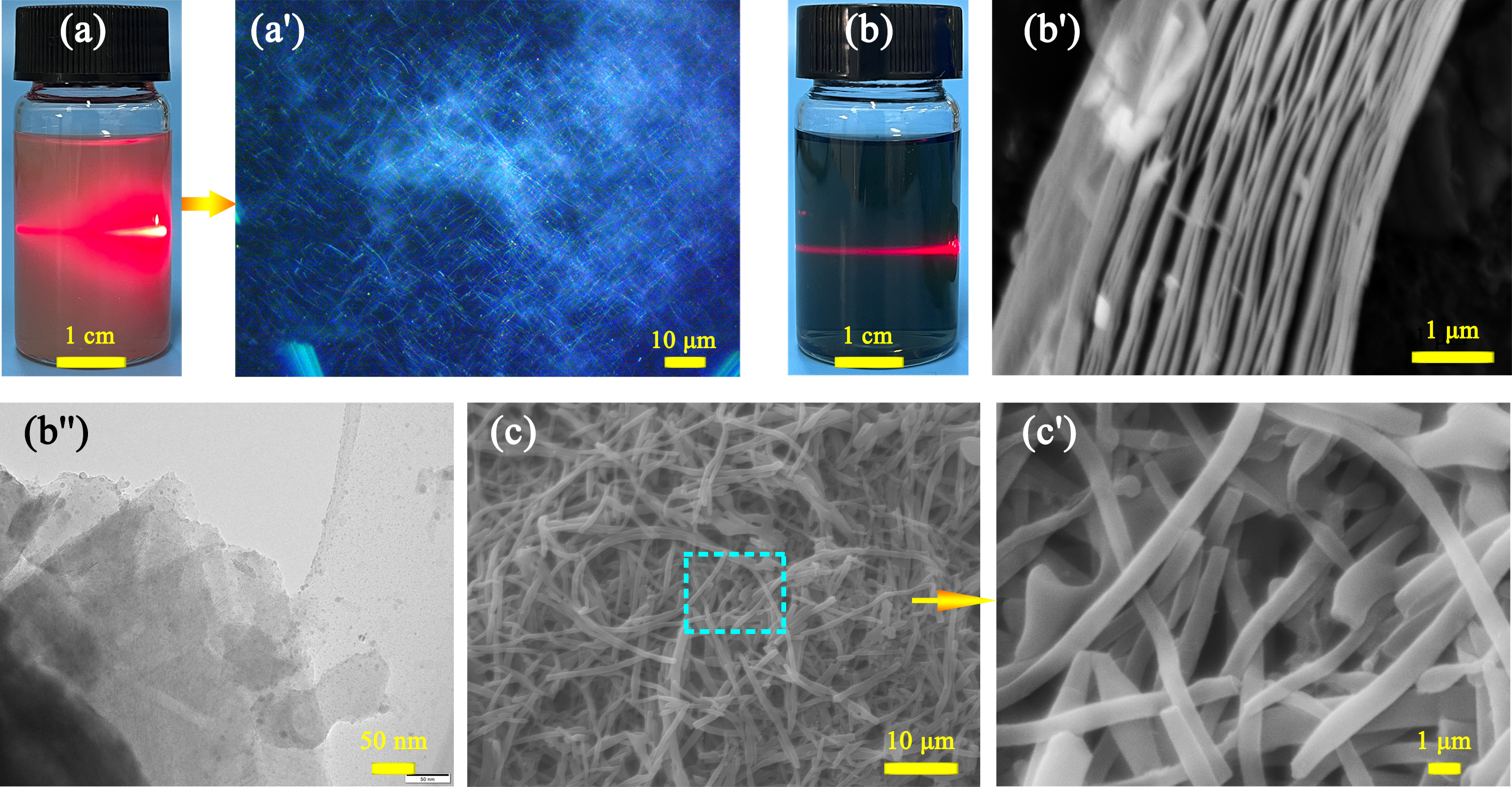


**Fig. S2 a** Optical photos of silver nanowire solution and **a'** microphotograph of the AgNW. **b** Optical photos of Ti_3_C_2_T_x_ MXene solution. **b'** SEM image of the multi-layered MXene. **b''** TEM images of the Ti_3_C_2_T_x_ MXene after ultrasonic dispersion. **c-c'** SEM images of the Pva-co-PE nanofibers


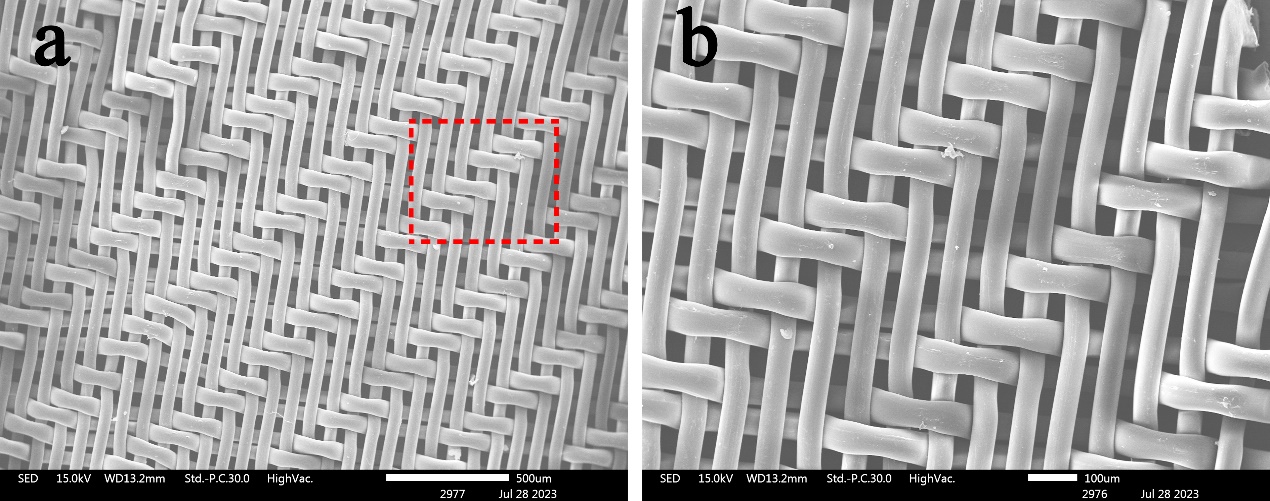


**Fig. S3** SEM images of twill structure on nylon fabric surface


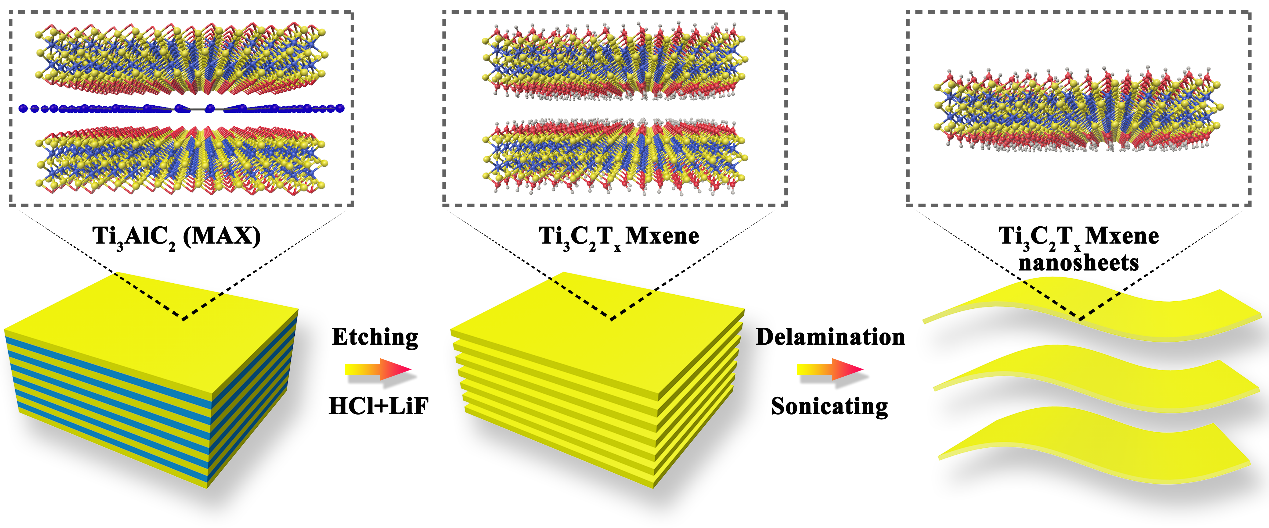


**Fig. S4** Synthesis of Ti_3_C_2_T_x_ MXene *via* the etching and delamination method


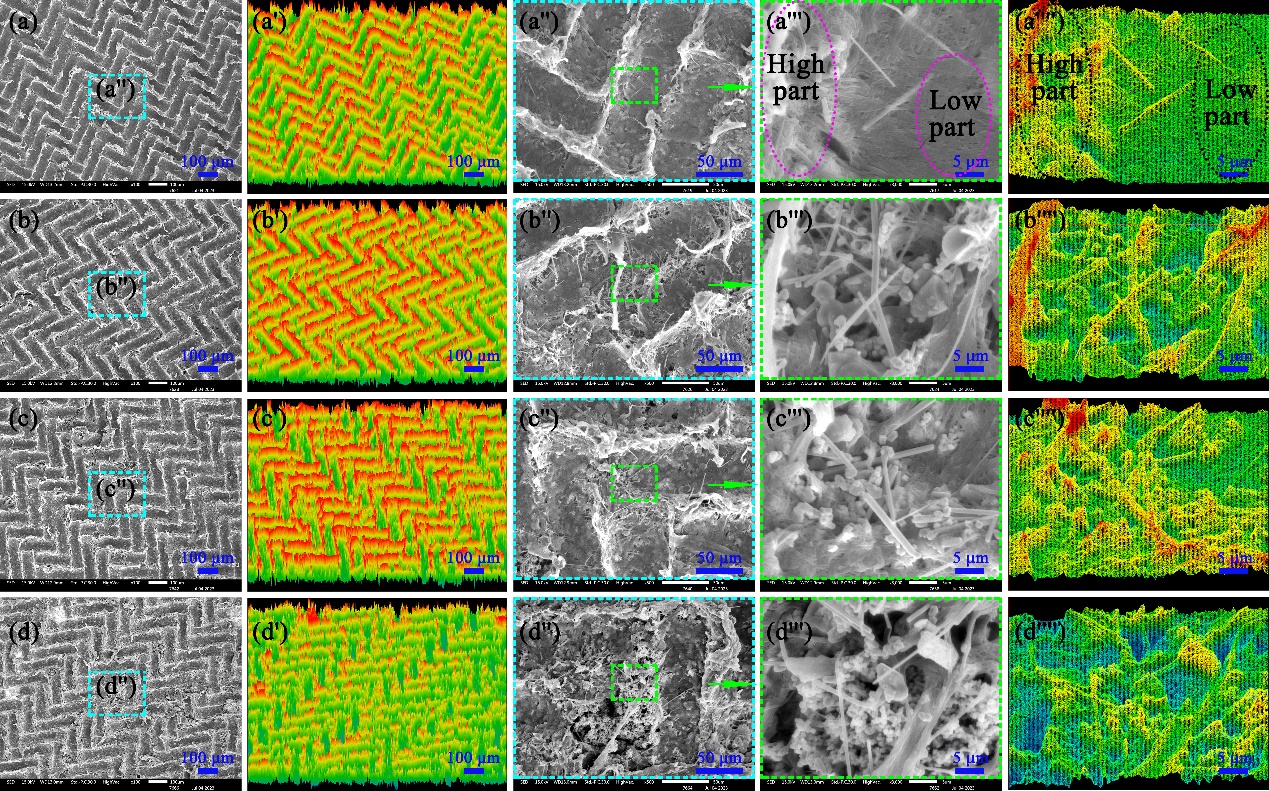


**Fig. S5** SEM image of surface morphology of the PM_x_Ag nanofiber composite membranes. **a-a''''** PM_1.6_Ag. **b-b''''** PM_2.2_Ag. **c-c''''** PM_5.2_Ag, and **d-d''''** PM_7.4_Ag


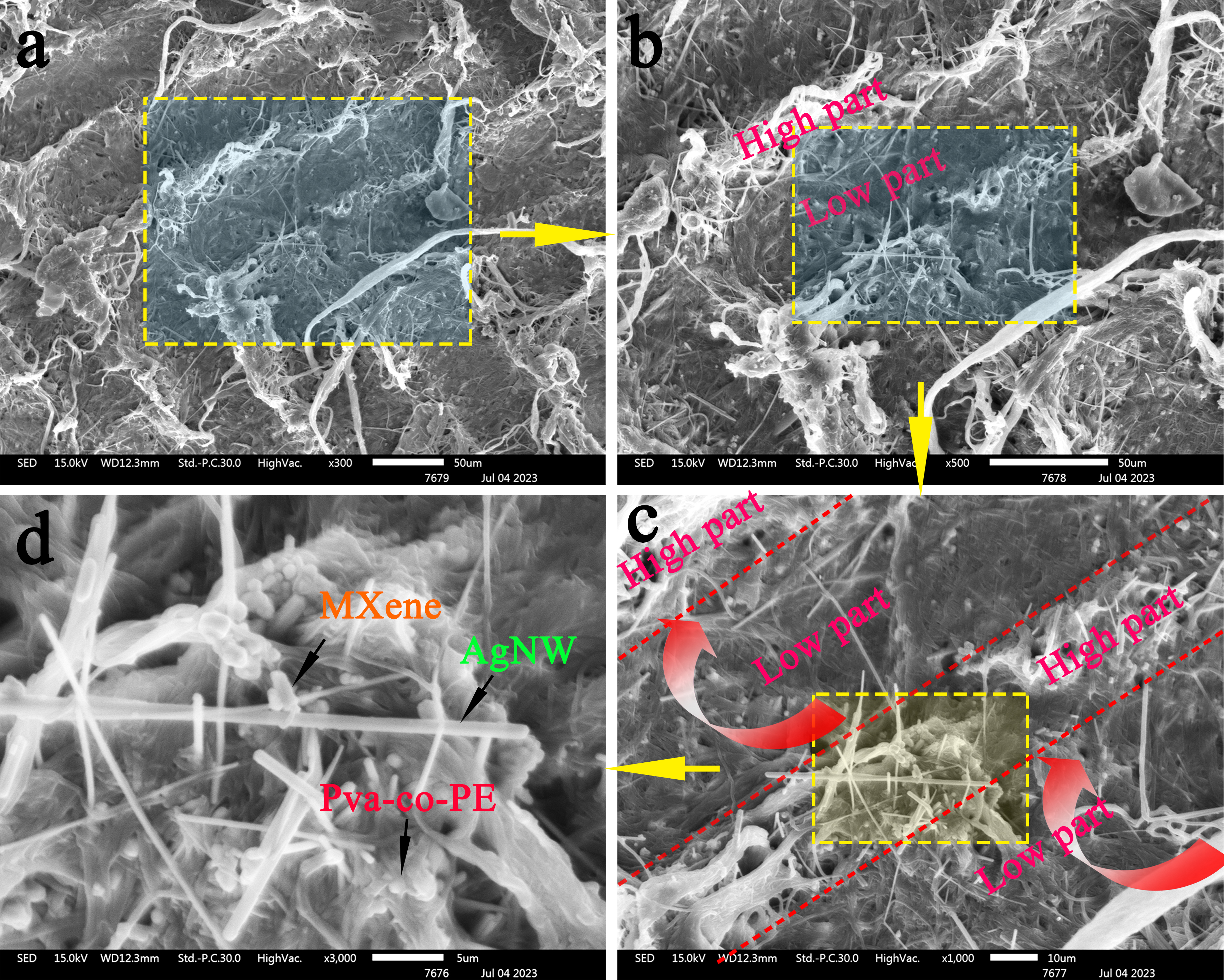


**Fig. S6** SEM images of surface morphology of the PM_5.2_Ag nanofiber composite membranes


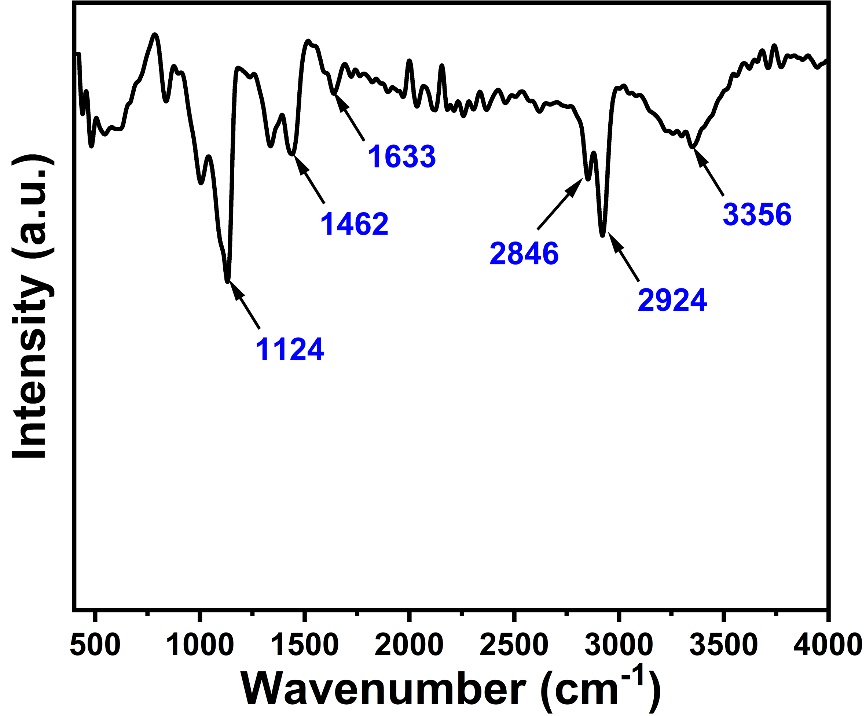


**Fig. S7** FTIR spectra of the Pva-co-PE membrane


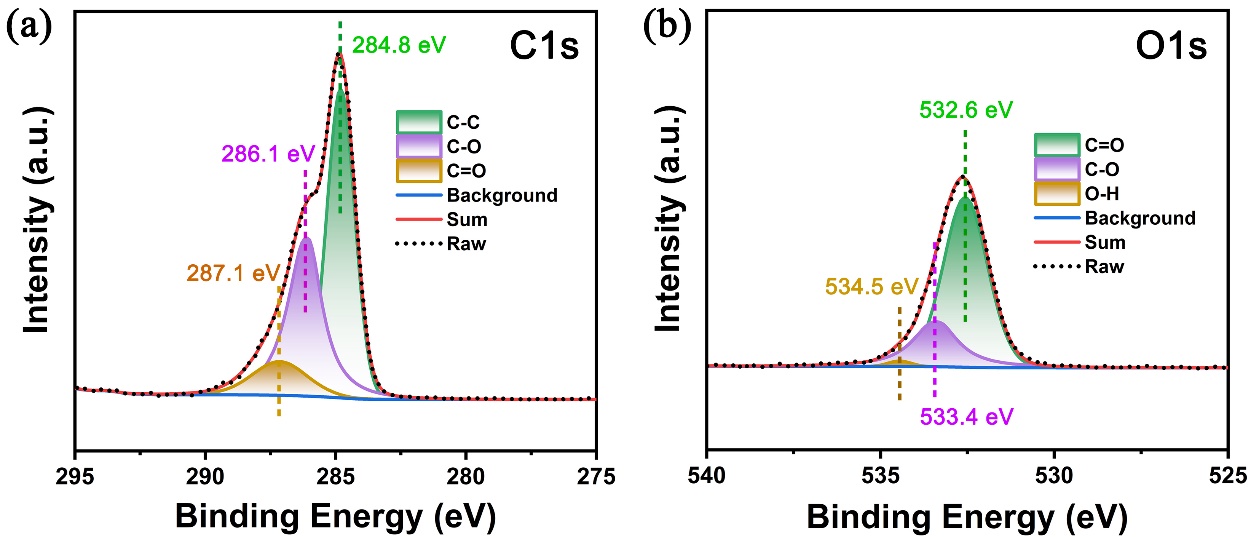


**Fig. S8** **a** High-resolution XPS spectra of C1s and **b** O1s for Pva-co-PE membrane
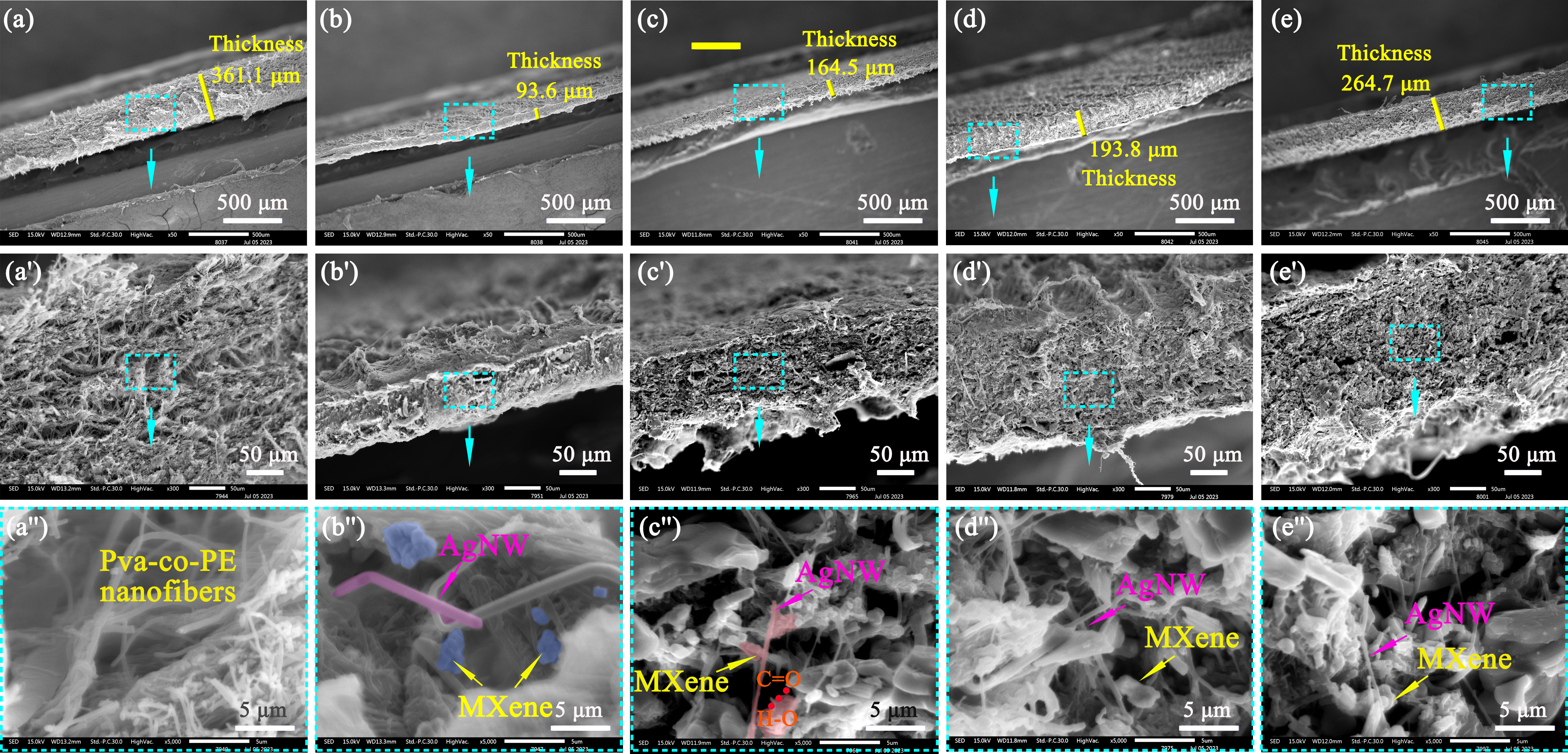


**Fig. S9** SEM images of cross-section morphologies of the Pva-co-PE and PM_x_Ag nanofiber composite membranes. a-a''' Pva-co-PE. b-b''' PM_1.6_Ag. **c-c'''** PM_2.2_Ag. **d-d'''** PM_5.2_Ag. **e-e'''** PM_7.4_Ag


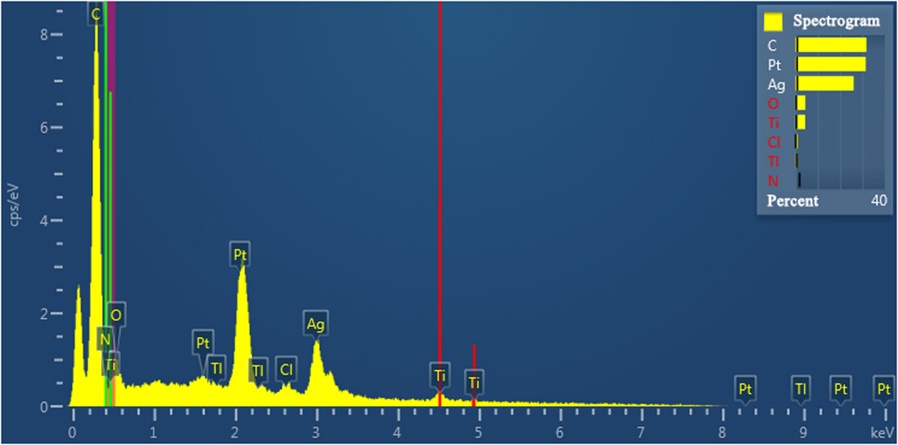


**Fig. S10** EDS spectra of the PM_2.2_Ag nanofiber composite membrane


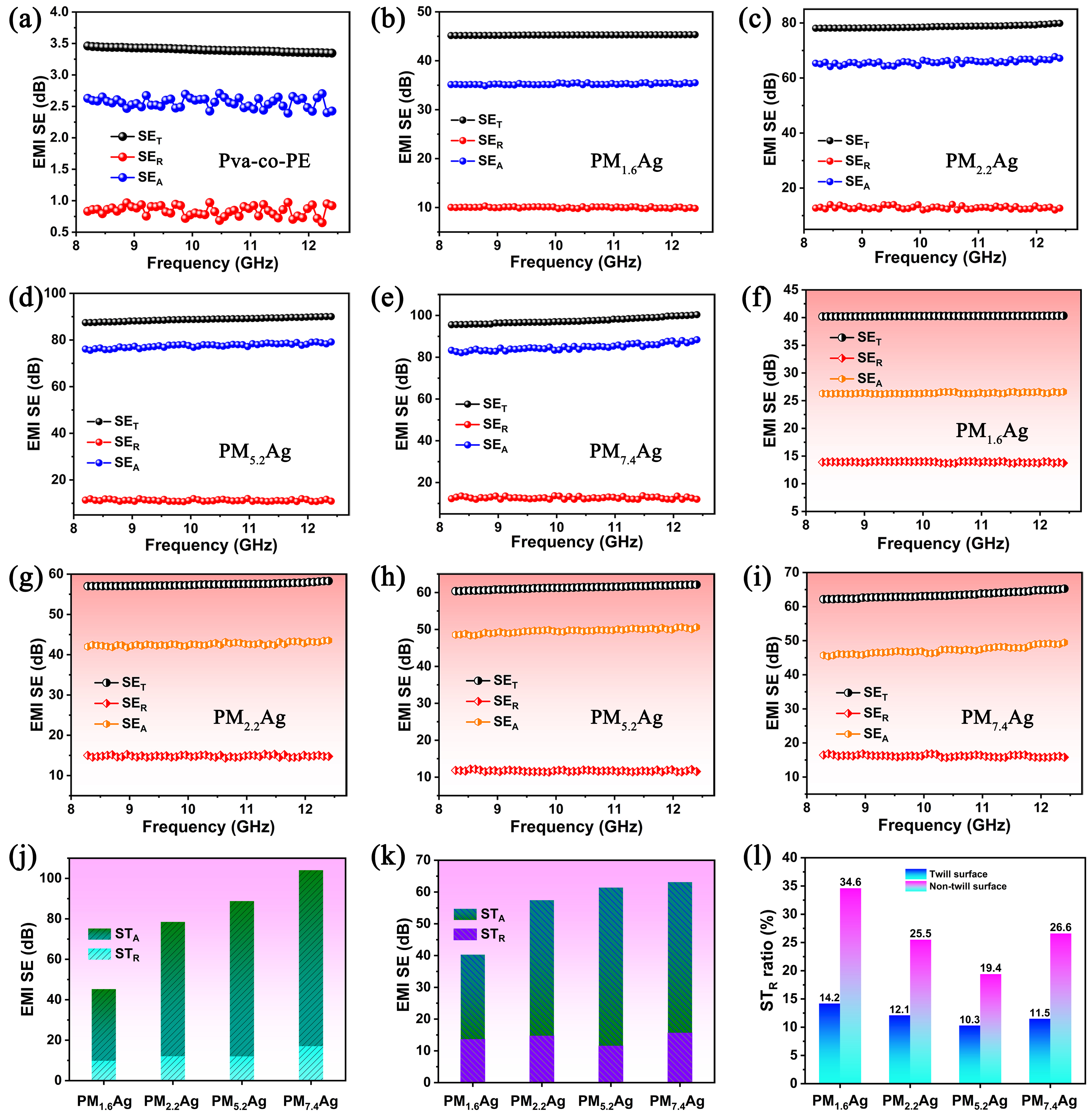


**Fig. S11** **a-e** EMI SE of Pva-co-PE and PM_x_Ag nanofiber composite membranes when EM wave incidence from twill surface. **f-i** EMI SE of PM_x_Ag nanofiber composite membrane when EM wave incidence from non-twill surface. **j** EMI ST_A_ and EMI ST_R_ of PM_x_Ag nanofiber composite membranes when EM wave incidence from twill surface. **k** ST_A_ and ST_R_ of PM_x_Ag nanofiber composite membranes when EM wave incidence from non-twill surface. **l** Comparison of the reflectance ratios of the EM wave at different incidence planes


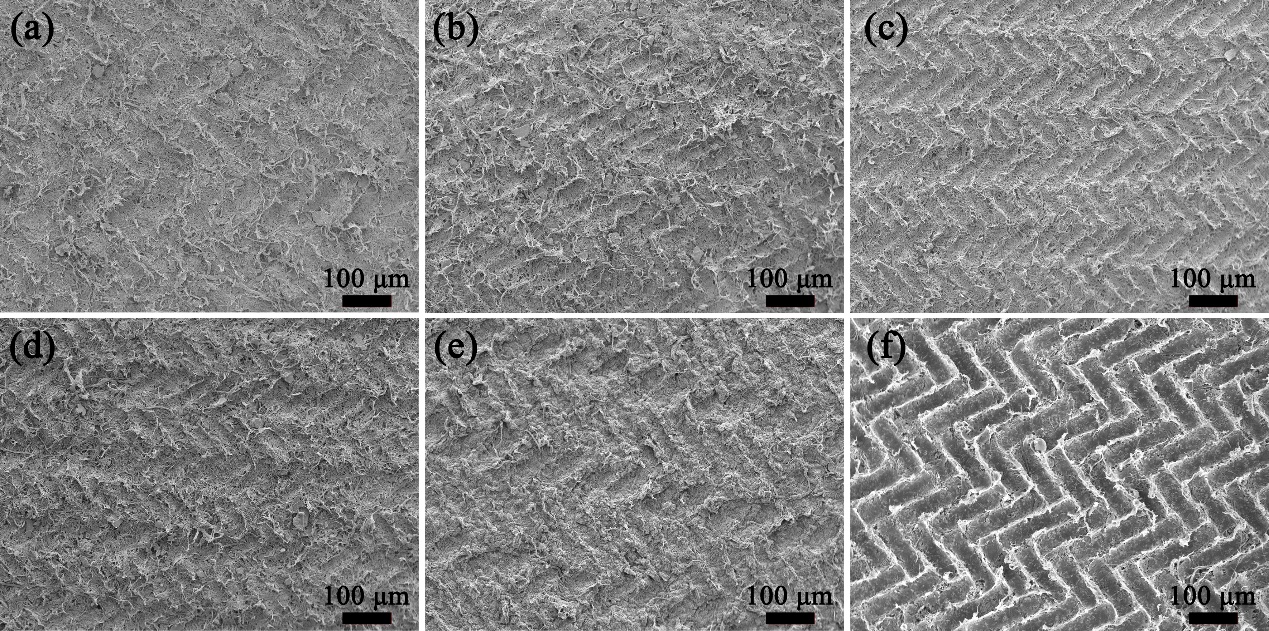


**Fig. S12** PM_x_Ag nanofiber composite membrane materials prepared by different pumping pressure: **a** 20 kPa. **b** 40 kPa. **c** 60 kPa. **d** 80 kPa. **e** 100 kPa. **f** 120 kPa


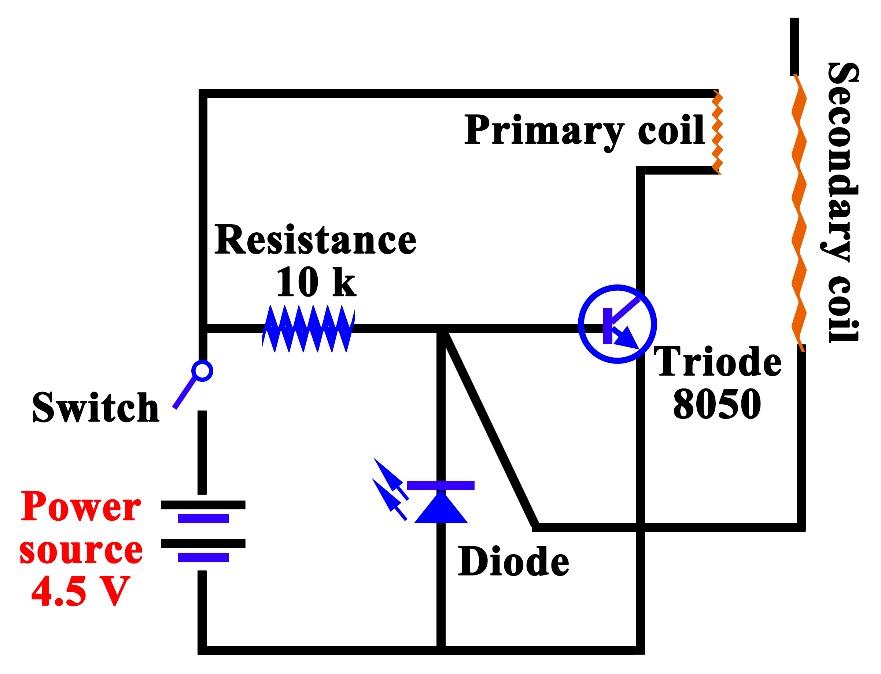


**Fig. S13** Circuit diagram of a Tesla coil device

**Table S1** Performance comparison of PM_x_Ag nanofiber membranes with previous reported EMI shielding materials

| **EMI shielding materials** | **Filling**  **Content (wt%)** | **Thickness**  **(μm)** | **Density**  **(g·cm^-3^)** | **EMI SE**  **(dB)** | **EMI SSE**  **(dB·cm^−1^)** | **EMI SSE/t**  **(dB·cm^2^·g^−1^)** | **Ref.** |  |
| --- | --- | --- | --- | --- | --- | --- | --- | --- |
| Pva-co-PE | 0 | 361.1 | 0.205 | / | / | / | **This work** |  |
| PM_1.6_Ag | 1.6 | 93.6 | 0.220 | 45.8 | 4893.2 | 22241.6 |  |  |
| PM_2.2_Ag | 2.2 | 164.5 | 0.239 | 78.9 | 4796.3 | 20068.2 |  |  |
| PM_5.2_Ag | 5.2 | 193.8 | 0.245 | 93.5 | 4824.5 | 19691.8 |  |  |
| PM_7.4_Ag | 7.4 | 264.7 | 0.253 | 103.9 | 3925.2 | 15514.6 |  |  |
| MXene/AgNWs/PVDF | 15 | 300 | 0.79 | 25.9 | 863.3 | 1091 | [S1] |  |
| FA-MXene/CNF | 30.5 | 120 | / | 63.9 | 5325 | / | [S2] |  |
| MXene/CNF film | 50 | 167 | 1.13 | 25 | 1497.0 | 884 | [S3] |  |
| MXene/PLA | / | 150 | / | 55.4 | 3693 | / | [S4] |  |
| MXene/CNF | 26 | 105 | / | 39 | 3714 | / | [S5] |  |
| MXene/AgNWs film | 20 | 120 | 1091 | 54.0 | 4500 | / | [S6] |  |
| MXene/FeCo/CNF | 55 | 340 | / | 58 | 1706 | / | [S7] |  |
| MXene/BC | 50 | 116 | / | 43.7 | 3767 | / | [S8] |  |
| MXene/Ni/PVDF | 20 | 100 | / | 19.5 | 1950 | / | [S9] |  |
| Ti_3_C_2_T*_x_*/Wax | / | 800 | 2.03 | 70 | 875 | 431 | [S10] |  |
| Ti_3_C_2_T*_x_*/PVA | 0.15 | 5000 | 0.011 | 28 | 56 | 5136 | [S11] |  |
| MXene | 100 | 11 | 0.001 | 68 | 28.4 | 25863 | [S12] |  |
| Graphene oxide/Fe_3_O_4_ paper | 50 | 300 | 0.78 | 24 | 800 | 1025.6 | [S13] |  |
| LPGF | 100 | 200 | 0.075 | 43.8 | 2190 | 29178 | [S14] |  |
| PPGF | 100 | 200 | 0.078 | 22 | 1100.0 | 14103 | [S14] |  |
| Ni@graphene/PVDF | 20 | 700 | / | 51.4 | 734 | / | [S15] |  |
| RGO/PEI | 1.38 | 2300 | 0.23 | 13 | 43.3 | 188 | [S16] |  |
| RGO/PU | 4.7 | 60000 | 6.01 | 57.7 | 1923 | 320 | [S17] |  |
| RGO/PS | 3.47 | 2500 | 0.25 | 45.1 | 41.9 | 167.5 | [S18] |  |
| RGO/PDMS | 0.36 | 1000 | 0.1 | 20 | 333.3 | 3333 | [S19] |  |
| RGO/WPU | 5 | 1000 | / | 34 | 33.8 | 338 | [S20] |  |
| RGO/Fe3O4 | 3.4 | 1800 | / | 13 | 8.9 | 49.5 | [S21] |  |
| RGO-γ-Fe2O3 | 2.3 | 360 | / | 20.3 | 15 | 416.7 | [S22] |  |
| RGO | 18.8 | / | / | 34.2 | 28.5 | 118.75 | [S23] |  |
| Graphene film | 50 | 10 | 1.49 | 43.8 | 43800 | 29396 | [S24] |  |
| Graphene film | / | 3.9 | 2.25 | 27.8 | 722078 | 320923 | [S25] |  |
| RGO/PMMA | 1.8 | 4000 | 0.79 | 19 | 47.4 | 60 | [S26] |  |
| Carbon nanotube/AgNWs/cellulose paper | 6.07 | 160 | 0.51 | 23.8 | 1487.5 | 2916.7 | [S27] |  |
| MWCNT/WPU | 76.2 | 100 | 0.04 | 21.1 | 211 | 5140 | [S28] |  |
| MWCNT/WPU | 76 | 320 | 0.45 | 49 | 1531 | 3408 | [S29] |  |
| MWCNT/SWCNT films | 30 | 130 | 0.8 | 65 | 500 | 625 | [S30] |  |
| MWCNT/WPU | 7.2 | 4500 | 0.126 | 50 | 111.1 | 881.8 | [S28] |  |
| MWCNT/PLLA | 1.47 | 2500 | 0.299 | 23 | 92 | 306.7 | [S31] |  |
| CNT/PS | 3.6 | 120 | 0.12 | 18.5 | 33 | 275 | [S32] |  |
| CNWs@G | 4.6 | 1600 | 0.159 | 36 | 370.7 | 2317 | [S33] |  |
| MWCNTs/Epoxy | 1.34 | 2000 | 0.2 | 40 | 20.1 | 100.5 | [S34] |  |
| CNTs/PC | 5 | 1850 | 0.185 | 25 | 20.8 | 112.6 | [S35] |  |
| CNTs/PP | 7.5 | 1000 | 0.1 | 35 | 37.2 | 372 | [S36] |  |
| SWCNTs/Pani | 15.5 | 2400 | 0.24 | 31.5 | 24.2 | 100.8 | [S23] |  |
| CNT/MLGEP | / | 1600 | 0.0089 | 47 | 293.8 | 32375 | [S37] |  |
| CNT/sponge | / | 2400 | 0.02 | 22 | 91.7 | 4583 | [S38] |  |
| MWCNT/CNF | / | 150 | 0.77 | 46.4 | 3093.3 | 4017.3 | [S39] |  |
| AgNWs/calcium alginate/PU film | 10 | 330 | 0.174 | 31.3 | 948.5 | 5451.1 | [S40] |  |
| AgNWs/PI | 4.5 | 5000 | 0.029 | 35 | 70 | 2416 | [S41] |  |
| AgNWs/PANI | 14 | 13 | / | 48 | 36923 | / | [S42] |  |
| AgNWs/cellulose papers | 0.53 | 160 | 0.53 | 48.6 | 3038 | 5585 | [S43] |  |
| Ag/Carbon fibers | 9 | 2500 | / | 38 | / | / | [S44] |  |
| AgNWs/PS | / | 800 | / | 33 | / | / | [S41] |  |
| AgNWs/Epoxy | / | 40 | / | 35 | / | / | [S45] |  |
| AgNWs/Epoxy | / | 13 | / | 50 | / | / | [S42] |  |


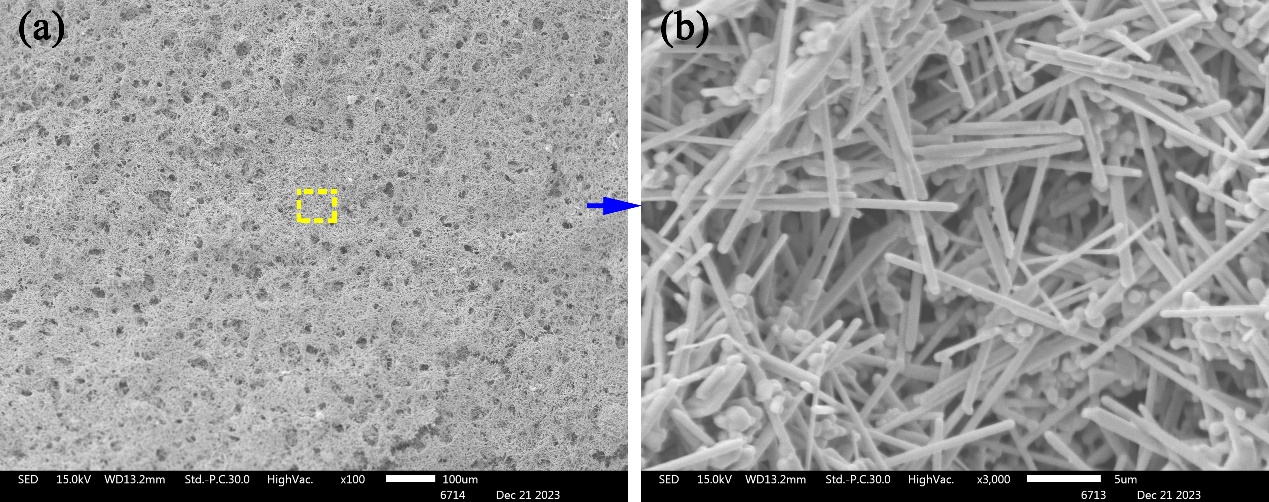


**Fig. S14** SEM images of the microstructure of PM_7.4_Ag nanofiber composite membrane after combustion


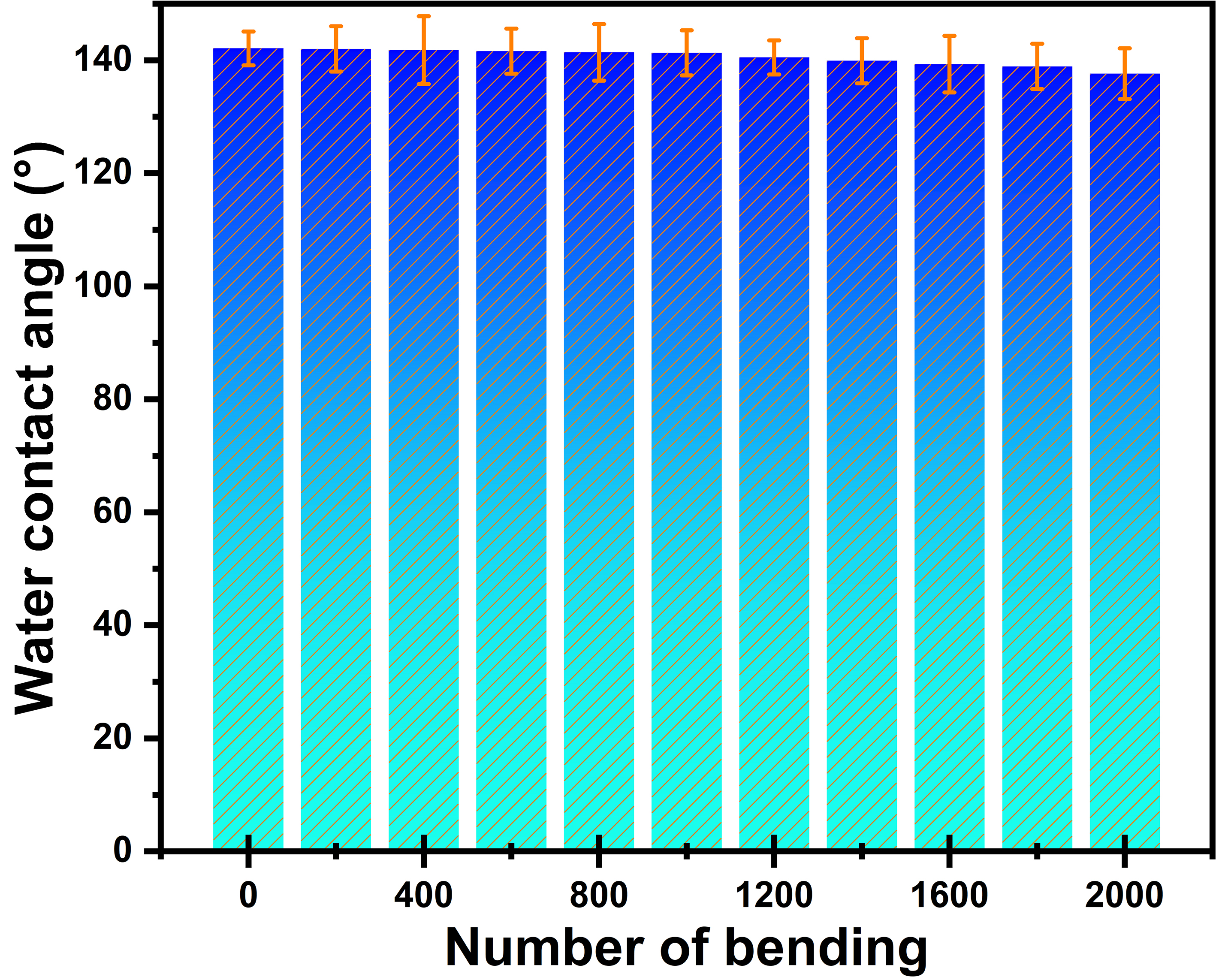


**Fig. S15** Water contact angle on the twill surface of the PM_7.4_Ag composite membrane after different bending times


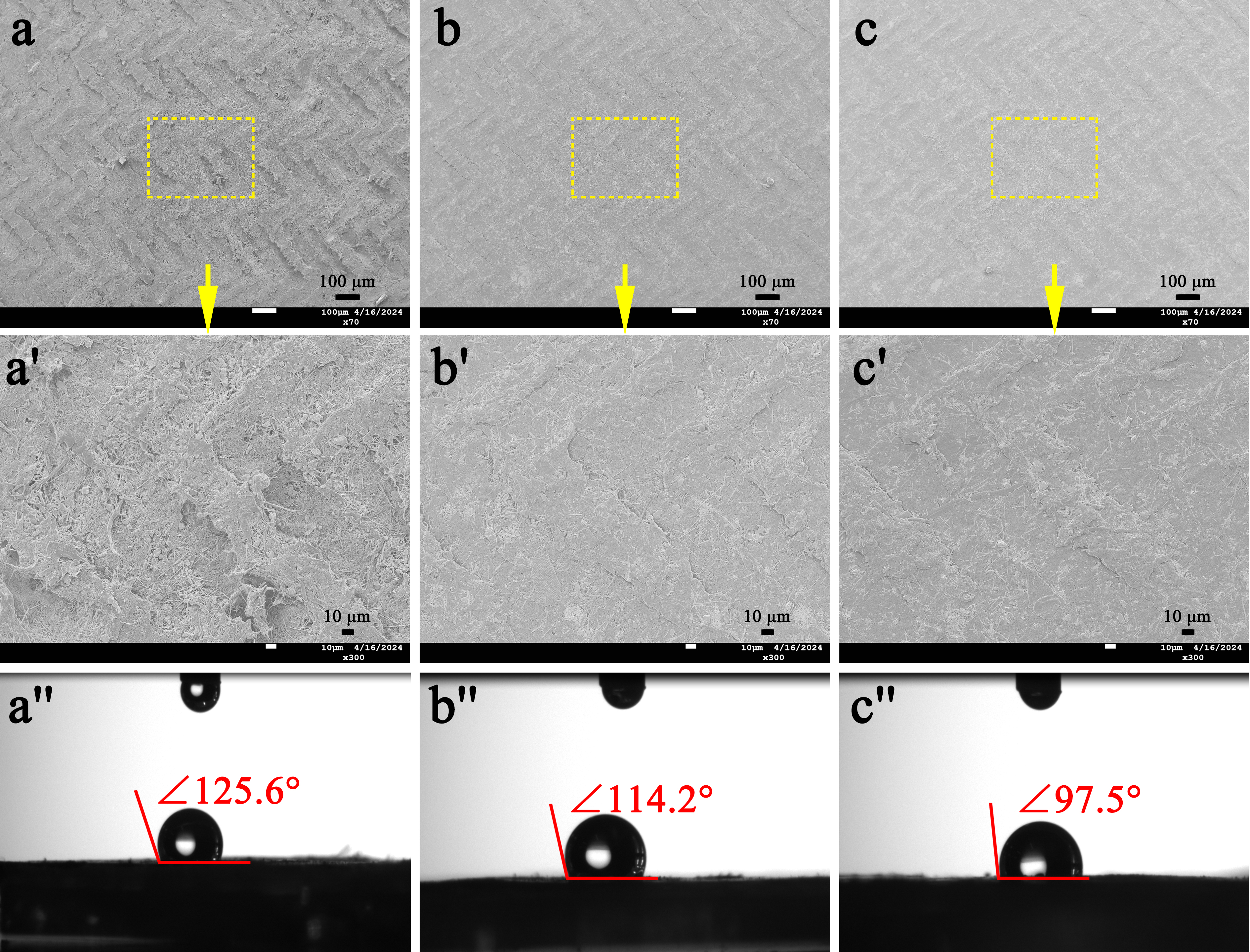


**Fig. S16** SEM images and water contact angle pictures of the PM_7.4_Ag composite membrane subjected to different compressive stresses, (**a, a',** **a''**) 2.5 MPa, (**b, b', b''**) 5.0 MPa, and (**c, c', c''**) 10.0 MPa


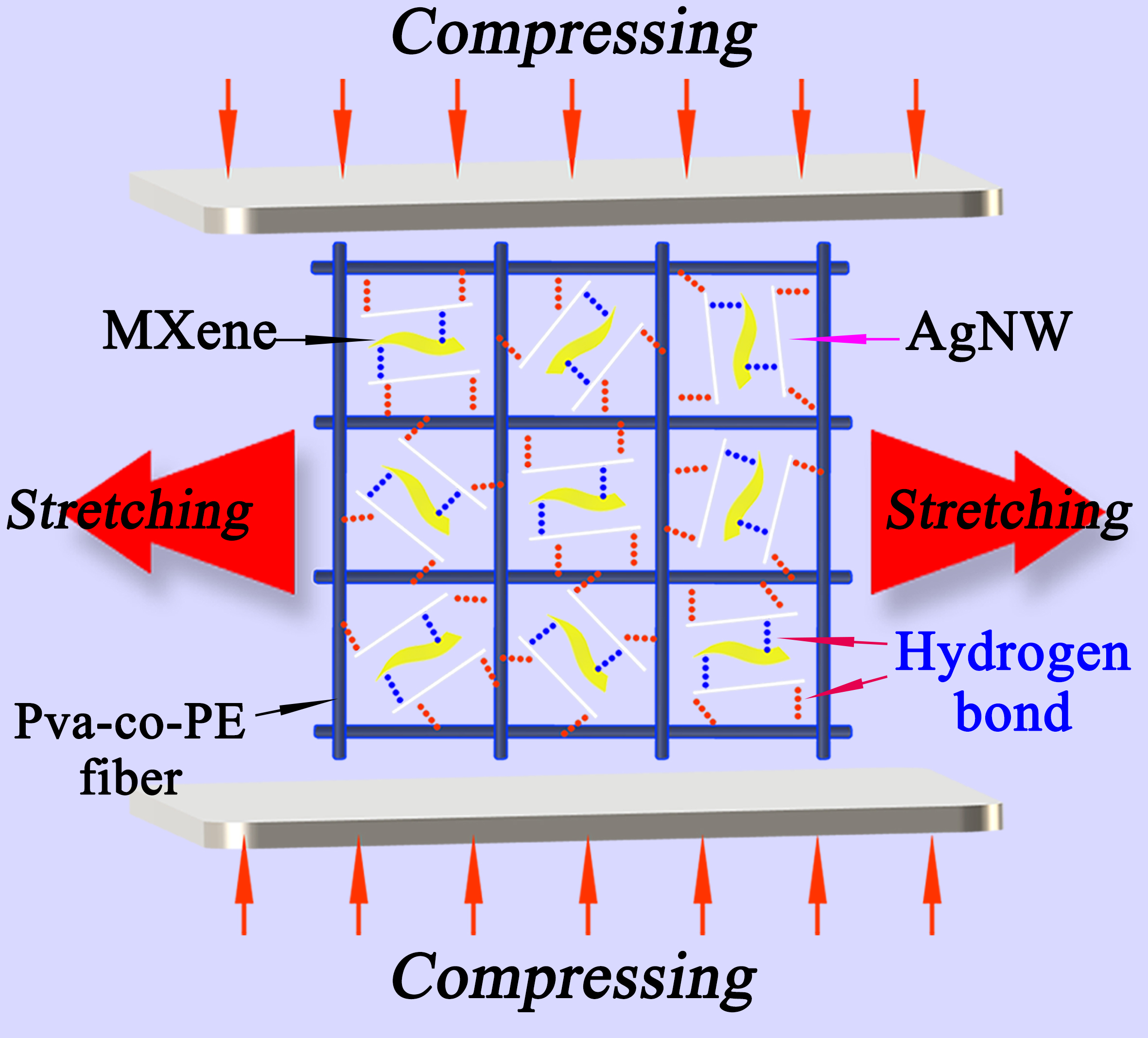


**Fig. S17** Stability diagram of the PM_x_Ag skeleton structure

**Supplementary References**

[S1] H. Cheng, Y. Pan, Q. Chen, R. Che, G. Zheng et al., Ultrathin flexible poly(vinylidene fluoride)/mxene/silver nanowire film with outstanding specific emi shielding and high heat dissipation. Adv. Composites Hybrid Mater. 4(3), 505-513 (2021). <https://doi.org/10.1007/s42114-021-00224-1>

[S2] Z. Guo, P. Ren, J. Wang, J. Tang, F. Zhang et al., Multifunctional sandwich-structured magnetic-electric composite films with joule heating capacities toward absorption-dominant electromagnetic interference shielding. Composites Part B: Engin. 236, 109836 (2022). <https://doi.org/10.1016/j.compositesb.2022.109836>

[S3] W.-T. Cao, F.-F. Chen, Y.-J. Zhu, Y.-G. Zhang, Y.-Y. Jiang et al., Binary strengthening and toughening of mxene/cellulose nanofiber composite paper with nacre-inspired structure and superior electromagnetic interference shielding properties. ACS Nano 12(5), 4583-4593 (2018). <https://doi.org/10.1021/acsnano.8b00997>

[S4] Z. Du, K. Chen, Y. Zhang, Y. Wang, P. He et al., Engineering multilayered mxene/electrospun poly(lactic acid) membrane with increscent electromagnetic interference (EMI) shielding for integrated joule heating and energy generating. Composites Commun. 26, 100770 (2021). <https://doi.org/10.1016/j.coco.2021.100770>

[S5] M. Ma, X. Liao, Q. Chu, S. Chen, Y. Shi et al., Construction of gradient conductivity cellulose nanofiber/mxene composites with efficient electromagnetic interference shielding and excellent mechanical properties. Composites Sci. Technol. 226, 109540 (2022). <https://doi.org/10.1016/j.compscitech.2022.109540>

[S6] L.-X. Liu, W. Chen, H.-B. Zhang, Q.-W. Wang, F. Guan et al., Flexible and multifunctional silk textiles with biomimetic leaf-like mxene/silver nanowire nanostructures for electromagnetic interference shielding, humidity monitoring, and self-derived hydrophobicity. Adv. Funct. Mater. 29(44), 1905197 (2019). <https://doi.org/10.1002/adfm.201905197>

[S7] M. Ma, W. Tao, X. Liao, S. Chen, Y. Shi et al., Cellulose nanofiber/mxene/feco composites with gradient structure for highly absorbed electromagnetic interference shielding. Chemical Engineering Journal. 452, 139471 (2023). <https://doi.org/10.1016/j.cej.2022.139471>

[S8] H. Liu, Z. Cui, L. Luo, Q. Liao, R. Xiong et al., Facile fabrication of flexible and ultrathin self-assembled Ti3C2Tx/bacterial cellulose composite films with multifunctional electromagnetic shielding and photothermal conversion performances. Chem. Engin. J. 454, 140288 (2023). <https://doi.org/10.1016/j.cej.2022.140288>

[S9] S.-J. Wang, D.-S. Li, L. Jiang, Synergistic effects between mxenes and ni chains in flexible and ultrathin electromagnetic interference shielding films. Adv. Mater. Interfaces 6(19), 1900961 (2019). <https://doi.org/10.1002/admi.201900961>

[S10] X. Li, X. Yin, S. Liang, M. Li, L. Cheng et al., 2d carbide mxene Ti2CTx as a novel high-performance electromagnetic interference shielding material. Carbon. 146, 210-217 (2019). <https://doi.org/10.1016/j.carbon.2019.02.003>

[S11] H. Xu, X. Yin, X. Li, M. Li, S. Liang et al., Lightweight ti2ctx mxene/poly(vinyl alcohol) composite foams for electromagnetic wave shielding with absorption-dominated feature. ACS Appl. Mater. Interfaces 11(10), 10198-10207 (2019). <https://doi.org/10.1021/acsami.8b21671>

[S12] F. Shahzad, M. Alhabeb, C.B. Hatter, B. Anasori, S. Man Hong et al., Electromagnetic interference shielding with 2d transition metal carbides (mxenes). Science 353(6304), 1137-1140 (2016). https://doi.org/10.1126/science.aag2421

[S13] W.-L. Song, X.-T. Guan, L.-Z. Fan, W.-Q. Cao, C.-Y. Wang et al., Magnetic and conductive graphene papers toward thin layers of effective electromagnetic shielding. J. Mater. Chem. A 3(5), 2097-2107 (2015). <https://doi.org/10.1039/C4TA05939E>

[S14] D. Lai, X. Chen, G. Wang, X. Xu, Y. Wang. Highly conductive porous graphene film with excellent folding resilience for exceptional electromagnetic interference shielding. J. Mater. Chem. C 8(26), 8904-8916 (2020). <https://doi.org/10.1039/D0TC01346C>

[S15] L. Liang, P. Xu, Y. Wang, Y. Shang, J. Ma et al., Flexible polyvinylidene fluoride film with alternating oriented graphene/ni nanochains for electromagnetic interference shielding and thermal management. Chem. Engin. J. 395, 125209 (2020). <https://doi.org/10.1016/j.cej.2020.125209>

[S16] J. Ling, W. Zhai, W. Feng, B. Shen, J. Zhang et al., Facile preparation of lightweight microcellular polyetherimide/graphene composite foams for electromagnetic interference shielding. ACS Appl. Mater. Interfaces 5(7), 2677-2684 (2013). <https://doi.org/10.1021/am303289m>

[S17] B. Shen, Y. Li, W. Zhai, W. Zheng. Compressible graphene-coated polymer foams with ultralow density for adjustable electromagnetic interference (emi) shielding. ACS Appl. Mater. Interfaces 8(12), 8050-8057 (2016). <https://doi.org/10.1021/acsami.5b11715>

[S18] D.X. Yan, H. Pang, B. Li, R. Vajtai, L. Xu et al., Structured reduced graphene oxide/polymer composites for ultra‐efficient electromagnetic interference shielding. Adv. Funct. Mater. 25(4), 559-566 (2015). <https://doi.org/10.1002/adfm.201403809>

[S19] Z. Chen, C. Xu, C. Ma, W. Ren, H.M. Cheng. Lightweight and flexible graphene foam composites for high‐performance electromagnetic interference shielding. Adv. Mater. 25(9), 1296-1300 (2013). <https://doi.org/10.1002/adma.201204196>

[S20] S.-T. Hsiao, C.-C. M. Ma, W.-H. Liao, Y.-S. Wang, S.-M. Li et al., Lightweight and flexible reduced graphene oxide/water-borne polyurethane composites with high electrical conductivity and excellent electromagnetic interference shielding performance. ACS Appl. Mater. Interfaces 6(13), 10667-10678 (2014). <https://doi.org/10.1021/am502412q>

[S21] K. Yao, J. Gong, N. Tian, Y. Lin, X. Wen et al., Flammability properties and electromagnetic interference shielding of pvc/graphene composites containing Fe3O4 nanoparticles. RSC Adv. 5(40), 31910-31919 (2015). <https://doi.org/10.1039/C5RA01046B>

[S22] B. Yuan, C. Bao, X. Qian, L. Song, Q. Tai et al., Design of artificial nacre-like hybrid films as shielding to mitigate electromagnetic pollution. Carbon 75, 178-189 (2014). <https://doi.org/10.1016/j.carbon.2014.03.051>

[S23] B. Yuan, L. Yu, L. Sheng, K. An, X. Zhao. Comparison of electromagnetic interference shielding properties between single-wall carbon nanotube and graphene sheet/polyaniline composites. J. Phys. D: Appl. Phys. 45(23), 235108 (2012). <https://doi.org/10.1088/0022-3727/45/23/235108>

[S24] Q. Wei, S. Pei, X. Qian, H. Liu, Z. Liu et al., Superhigh electromagnetic interference shielding of ultrathin aligned pristine graphene nanosheets film. Adv. Mater. 32(14), 1907411 (2020). <https://doi.org/10.1002/adma.201907411>

[S25] T. Zhou, C. Xu, H. Liu, Q. Wei, H. Wang et al., Second time-scale synthesis of high-quality graphite films by quenching for effective electromagnetic interference shielding. ACS Nano 14(3), 3121-3128 (2020). <https://doi.org/10.1021/acsnano.9b08169>

[S26] H.-B. Zhang, Q. Yan, W.-G. Zheng, Z. He, Z.-Z. Yu. Tough graphene−polymer microcellular foams for electromagnetic interference shielding. ACS Appl. Mater. Interfaces 3(3), 918-924 (2011). <https://doi.org/10.1021/am200021v>

[S27] H. Y. Choi, T.-W. Lee, S.-E. Lee, J. Lim, Y. G. Jeong. Silver nanowire/carbon nanotube/cellulose hybrid papers for electrically conductive and electromagnetic interference shielding elements. Composites Sci. Technol. 150, 45-53 (2017). <https://doi.org/10.1016/j.compscitech.2017.07.008>

[S28] Z. Zeng, H. Jin, M. Chen, W. Li, L. Zhou et al., Lightweight and anisotropic porous mwcnt/wpu composites for ultrahigh performance electromagnetic interference shielding. Adv. Funct. Mater. 26(2), 303-310 (2016). <https://doi.org/10.1002/adfm.201503579>

[S29] Z. Zeng, M. Chen, H. Jin, W. Li, X. Xue et al., Thin and flexible multi-walled carbon nanotube/waterborne polyurethane composites with high-performance electromagnetic interference shielding. Carbon 96, 768-777 (2016). <https://doi.org/10.1016/j.carbon.2015.10.004>

[S30] S. Lu, J. Shao, K. Ma, D. Chen, X. Wang et al., Flexible, mechanically resilient carbon nanotube composite films for high-efficiency electromagnetic interference shielding. Carbon 136, 387-394 (2018). <https://doi.org/10.1016/j.carbon.2018.04.086>

[S31] T. Kuang, L. Chang, F. Chen, Y. Sheng, D. Fu et al., Facile preparation of lightweight high-strength biodegradable polymer/multi-walled carbon nanotubes nanocomposite foams for electromagnetic interference shielding. Carbon 105, 305-313 (2016). <https://doi.org/10.1016/j.carbon.2016.04.052>

[S32] Y. Yang, M.C. Gupta, K.L. Dudley, R.W. Lawrence, Novel carbon nanotube−polystyrene foam composites for electromagnetic interference shielding. Nano Lett. 5(11), 2131-2134 (2005). <https://doi.org/10.1021/nl051375r>

[S33] L. Kong, X. Yin, M. Han, X. Yuan, Z. Hou et al., Macroscopic bioinspired graphene sponge modified with in-situ grown carbon nanowires and its electromagnetic properties. Carbon 111, 94-102 (2017). <https://doi.org/10.1016/j.carbon.2016.09.066>

[S34] Y. Chen, H.-B. Zhang, Y. Yang, M. Wang, A. Cao et al., High-performance epoxy nanocomposites reinforced with three-dimensional carbon nanotube sponge for electromagnetic interference shielding. Adv. Funct. Mater. 26, 447–455 (2016). <https://doi.org/10.1002/adfm.201503782>

[S35] M. Arjmand, M. Mahmoodi, G.A. Gelves, S. Park, U. Sundararaj Electrical and electromagnetic interference shielding properties of flow-induced oriented carbon nanotubes in polycarbonate. Carbon 49(11), 3430-3440 (2011). <https://doi.org/10.1016/j.carbon.2011.04.039>

[S36] M.H. Al-Saleh, U. Sundararaj Electromagnetic interference shielding mechanisms of CNT/polymer composites. Carbon 47, 1738–1746 (2009). <https://doi.org/10.1016/j.carbon.2009.02.030>

[S37] Q. Song, F. Ye, X. Yin, W. Li, H. Li et al., Carbon nanotube–multilayered graphene edge plane core–shell hybrid foams for ultrahigh-performance electromagnetic-interference shielding. Adv. Mater. 29, 1701583 (2017). <https://doi.org/10.1002/adma.201701583>

[S38] M. Crespo, M. González, A. L. Elías, L. Pulickal Rajukumar, J. Baselga, M. Terrones, J. Pozuelo. Ultra-light carbon nanotube sponge as an efficient electromagnetic shielding material in the ghz range. physica status solidi (RRL) – Rapid Research Letters. 8(8), 698-704 (2014). <https://doi.org/10.1002/pssr.201409151>

[S39] H. Zhang, X. Sun, Z. Heng, Y. Chen, H. Zou et al., Robust and flexible cellulose nanofiber/multiwalled carbon nanotube film for high-performance electromagnetic interference shielding. Ind. Eng. Chem. Res. 57, 17152–17160 (2018). <https://doi.org/10.1021/acs.iecr.8b04573>

[S40] L.-C. Jia, D.-X. Yan, X. Liu, R. Ma, H.-Y. Wu et al., Highly efficient and reliable transparent electromagnetic interference shielding film. ACS Appl. Mater. Interfaces 10, 11941–11949 (2018). <https://doi.org/10.1021/acsami.8b00492>

[S41] J. Ma, K. Wang, M. Zhan A comparative study of structure and electromagnetic interference shielding performance for silver nanostructure hybrid polyimide foams. RSC Adv. 5, 65283–65296 (2015). <https://doi.org/10.1039/c5ra09507g>

[S42] F. Fang, Y.-Q. Li, H.-M. Xiao, N. Hu, S.-Y. Fu. Layer-structured silver nanowire/polyaniline composite film as a high performance x-band EMI shielding material. J. Mater. Chem. C 4(19), 4193-4203 (2016). <https://doi.org/10.1039/C5TC04406E>

[S43] T.-W. Lee, S.-E. Lee, Y.G. Jeong Highly effective electromagnetic interference shielding materials based on silver nanowire/cellulose papers. ACS Appl. Mater. Interfaces 8, 13123–13132 (2016). <https://doi.org/10.1021/acsami.6b02218>

[S44] J. Li, S. Qi, M. Zhang, Z. Wang Thermal conductivity and electromagnetic shielding effectiveness of composites based on Ag-plating carbon fiber and epoxy. J. Appl. Polym. Sci. 132, 42306 (2015). <https://doi.org/10.1002/app.42306>

[S45] N.M. Abbasi, H. Yu, L. Wang, Zain-ul-Abdin, W.A. Amer et al., Preparation of silver nanowires and their application in conducting polymer nanocomposites. Mater. Chem. Phys. 166, 1–15 (2015). <https://doi.org/10.1016/j.matchemphys.2015.08.056>
